# Reduced Gut Microbiome Diversity and Metabolome Differences in Rhinoceros Species at Risk for Iron Overload Disorder

**DOI:** 10.3389/fmicb.2019.02291

**Published:** 2019-10-04

**Authors:** Terri L. Roth, Alexandra Switzer, Miki Watanabe-Chailland, Elisabeth M. Bik, David A. Relman, Lindsey E. Romick-Rosendale, Nicholas J. Ollberding

**Affiliations:** ^1^Center for Conservation and Research of Endangered Wildlife, Cincinnati Zoo & Botanical Garden, Cincinnati, OH, United States; ^2^Department of Medicine, School of Medicine, Stanford University, Stanford, CA, United States; ^3^Department of Microbiology and Immunology, School of Medicine, Stanford University, Stanford, CA, United States; ^4^Division of Pathology and Laboratory Medicine, Cincinnati Children’s Hospital Medical Center, Cincinnati, OH, United States; ^5^Infectious Diseases Section, VA Palo Alto Health Care System, Palo Alto, CA, United States; ^6^Division of Biostatistics and Epidemiology, Cincinnati Children’s Hospital Medical Center, Cincinnati, OH, United States; ^7^Department of Pediatrics, College of Medicine, University of Cincinnati, Cincinnati, OH, United States

**Keywords:** black rhinoceros, Sumatran rhinoceros, rhinoceros, iron overload, microbiome, metabolome, microbial diversity, disease susceptibility

## Abstract

Iron overload disorder (IOD) affects many wildlife species cared for *ex situ*. Two of the four rhinoceros species in human care, Sumatran rhinoceros (*Dicerorhinus sumatrensis*) and black rhinoceros (*Diceros bicornis*), are susceptible, whereas the other two, white rhinoceros (*Ceratotherium simum*) and greater one-horned (GOH) rhinoceros (*Rhinoceros unicornis*), are relatively resistant to IOD. Complex interrelationships exist between mammalian hosts, their indigenous gut microbiota, metabolome, physical condition, and iron availability. The goal of this study was to gain insight into these relationships within the family Rhinocerotidae. Specific objectives were to (1) characterize the gut microbiome and metabolome of four rhinoceros species; (2) compare the microbiome and metabolome of IOD-susceptible and IOD-resistant rhinoceros species; and (3) identify variation in the microbiome and metabolome associated with compromised health or disease in IOD-susceptible rhinoceroses. Fecal samples were collected from 31 rhinoceroses (Sumatran rhinoceros, *n* = 3; black rhinoceros, *n* = 6; GOH rhinoceros, *n* = 9; white rhinoceros, *n* = 13) located at five facilities, and matched fecal aliquots were processed for microbiome and metabolome analyses using 16S rRNA gene sequencing and nuclear magnetic resonance spectroscopy, respectively. Despite the phylogenetic disparity and dissimilar zoo diets of the hosts, the structure of the fecal microbiota of the two IOD-susceptible rhinoceros species were more closely related to each other than to those of the two IOD-resistant species (Bray–Curtis dissimilarity; IOD-susceptible vs. IOD-resistant *p*-value < 0.001). In addition, IOD-susceptible rhinoceroses exhibited less microbial diversity than their IOD-resistant relatives (Shannon diversity; *p*-value < 0.001) which could have health implications. Of note, the black rhinoceros was distinct among the four rhinoceros species with the most divergent fecal metabolome; interestingly, it contained higher concentrations of short chain fatty acids. Neither age nor sex were associated with differences in microbial community composition (*p* = 0.253 and 0.488, respectively) or fecal metabolomic profile (*p* = 0.634 and 0.332, respectively). Differences in the distal gut microbiomes between IOD-resistant and IOD-susceptible rhinoceroses support hypotheses that gut microbes play a role in host iron acquisition, and further studies and experiments to test these hypotheses are warranted.

## Introduction

**FIGURE 1:**
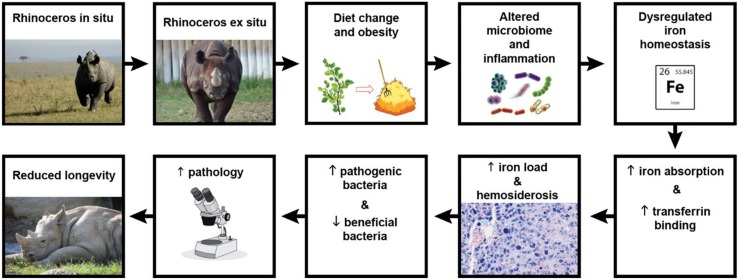
Schematic of the study’s suggested hypothetical model. When black rhinoceroses are captured, their diet changes from browse to hay, altering their natural microbiome and subsequent nutrient absorption. Additionally, the high nutrient diet and lack of exercise lead to over conditioning, obesity, inflammation, and metabolic disturbance which results in dysregulated iron homeostasis. Transferrin binding sites become saturated with iron which is also increasingly stored in organ tissues (hemosiderosis). Microbial competition in an iron-rich environment alters gut colonization in favor of pathogenic lineages, while more beneficial populations decrease. The resulting microbial and metabolic disturbances lead to greater disease susceptibility, health syndromes and pathologies, ultimately reducing the population’s median longevity. (Photo credits following the flow of the diagram: Renee Fulconis, Cincinnati Zoo & Botanical Garden; Hailee Butler, Cincinnati Zoo & Botanical Garden.)

Iron overload disease has been identified in a wide range of wildlife species maintained *ex situ* including birds, tapirs, primates, bats, dolphins, pinnipeds, rodents, rhinoceroses, and lagomorphs (reviewed by Clauss and Paglia, 2012). In the rhinoceros, the term iron overload disorder (IOD) was adopted in 2012 to describe the condition of excess body iron identified post-mortem in the form of excessive organ tissue hemosiderosis in this taxon, and to catalyze efforts to better understand the frequency of progression to disease-states and its role in predisposing individuals to unrelated diseases (Dennis et al., 2012). IOD affects two of the four rhinoceros species maintained in zoos, the black rhinoceros (*Diceros bicornis*) (Olias et al., 2012; Paglia and Tsu, 2012) and the Sumatran rhinoceros (*Dicerorhinus sumatrensis*) (Paglia and Tsu, 2012; Roth et al., 2017). Both species are browsers in the wild with diets consisting primarily of shrubs, bushes, leaves, twigs, tree branches, and bark (Hall-Martin et al., 1982; Van Strien, 1986). In contrast, wild white rhinoceros (*Ceratotherium simum*) and greater one-horned (GOH) rhinoceros (*Rhinoceros unicornis*) are grazers with grasses making up 100% and over 85% of their diets, respectively (Shrader et al., 2006; Hazarika and Saikia, 2012), and IOD is only rarely reported in these species (Ball and Mueller, 2011; Olias et al., 2012). Because the food of browsers is more difficult to mimic in zoos than that of grazers, suboptimal browser diets have always been suspected to be responsible for IOD in the two IOD-susceptible rhinoceros species. For certain mammalian species, there is some evidence that maintaining a low iron diet offers a practical way to prevent disease progression (Clauss and Paglia, 2012). However, if caretakers follow recommended nutritional guidelines, rhinoceros diets already are inherently low in bioavailable iron so there is limited ability for further reduction. Moreover, dietary iron may not be the primary cause of disease progression. The physiological sequelae resulting from dietary and life-style differences experienced by zoo animals vs. those in the wild may also lead to IOD (Figure 1). For example, black rhinoceroses maintained in zoos have elevated biomarkers of inflammation and decreased insulin sensitivity relative to wild counterparts, suggesting metabolic disturbance (Schook et al., 2015). The analogous condition in obese humans is associated with hyperferritinemia, metabolic changes and even hemosiderosis in some cases (Aigner et al., 2015; Datz et al., 2017).

High iron stores are thought to be associated with heightened infectious disease susceptibility in black rhinoceroses, however, the mechanism remains obscure (Dennis et al., 2012). Although iron stores in the form of hemosiderosis are noted in many tissues of most black rhinoceroses at necropsy (Olias et al., 2012; Paglia and Tsu, 2012), hemochromatosis is seldom reported as the cause of death in this species (Dennis et al., 2007; Olias et al., 2012; Roth et al., 2019). A compilation of United States zoo mortality data over a 16-year period ending in 2007, revealed that 22% of black rhinoceros deaths were due to infectious disease including salmonellosis and tuberculosis (Roth et al., 2009), both of which are more severe in an environment with increased iron availability (Gobin and Horwitz, 1996; Kortman et al., 2012). Over the past decade, 18% of reported black rhinoceros deaths were due to infectious or liver disease (Roth et al., 2019). Both contribute to low median longevity (19 years) experienced by this species when compared to the GOH rhinoceros and white rhinoceros (>30 years) (Survival Statistics Library, 2019). In contrast, gastrointestinal tract disease is more prevalent in white rhinoceroses, and infectious disease is rare (Roth et al., 2019).

To advance our understanding of IOD in the rhinoceros, it is important to clarify the complex relationship between the host, its indigenous microbial populations and iron availability. The mammalian gut is colonized at birth by bacteria which play an integral role in host nutrition, metabolic regulation and immunology (reviewed in Gerritsen et al., 2011). Most of these bacteria require iron for survival, function and proliferation (Andrews et al., 2003). To maintain the delicate balance of a healthy gut microbiota without excessive pathogen proliferation, the body limits the availability of iron to microbes by ensuring free iron is rapidly and tightly bound by host proteins. The gut microbiota competes for iron, and therefore iron availability directly affects population structure (Tompkins et al., 2001; Werner et al., 2011; Dostal et al., 2014). Iron also can enhance microbial virulence (Kortman et al., 2012). In contrast, iron-independent taxa are largely beneficial barrier microbes like those found in probiotics (Weinberg, 1997; Morelli, 2000; Madden and Hunter, 2002) and are out-competed in an iron-rich environment. These interdependencies explain why iron supplementation during an infection often exacerbates the problem (Griffiths, 1999; Zimmermann et al., 2010). In fact, there have been several studies on humans and mice linking the effects of iron overload, iron supplementation and iron insufficiency on the gut microbiome to the physiological health of the individual (Kelly et al., 1987; Tompkins et al., 2001; Zimmermann et al., 2010).

It is unknown if there are changes in the microbiota associated with IOD in rhinoceroses, and if so, whether these changes are a contributor to, or sequelum of, the disease. Since the gut microbiota of rodents and humans has been shown to affect absorption of nutrients, including iron (Reddy et al., 1972; González et al., 2017) and iron-related proteins (Deschemin et al., 2016), it is possible that dysbiosis could directly affect iron availability. Alternatively, microbial community shifts could help explain IOD etiology if they are characterized by the proliferation of pathogens, or loss of beneficial taxa.

The gut microbiota produces metabolites which also play a vital role in organismal health (Flint et al., 2015). For example, short chain fatty acids (SCFAs) produced by microbes from non-digestible carbohydrates are believed to have anti-inflammatory and anti-carcinogenic activity (Hosseini et al., 2011; Vinolo et al., 2011). They also tend to be elevated in the fecal metabolome of obese humans (Patil et al., 2012; Ríos-Covián et al., 2016). It has already been demonstrated that the serum metabolic profile of Sumatran rhinoceroses with symptomatic hemochromatosis differs from that of healthy individuals (Watanabe et al., 2016). However, it is not known if such changes occur as a precursor to or an effect of illness. Serum metabolites may be largely host-generated, whereas the by-products of microbial populations in the gut are better represented in the fecal metabolome (Karu et al., 2018). It is therefore of interest to characterize the fecal metabolome of rhinoceroses to determine if the byproducts of the gut microbiome change in association with IOD.

To shed some light on these complex relationships, the goal of this study was to compare the gut microbiome and metabolome of IOD-susceptible and IOD-resistant rhinoceroses cared for in zoos. The hypothesis was that the microbiome and metabolome of the two IOD-susceptible rhinoceros species would differ from those of the two IOD-resistant rhinoceros species. Specific objectives were to: (1) characterize the gut microbiome and metabolome of four rhinoceros species, (2) compare the microbiome and metabolome of IOD-susceptible to IOD-resistant rhinoceros species, and (3) identify microbiota and metabolomic differences associated with compromised health or disease in IOD-susceptible rhinoceroses.

## Materials and Methods

### Ethics Statement

The Institutional Animal Care and Use Committee of the Cincinnati Zoo & Botanical Garden deemed that, in accordance with the United States Animal Welfare Act, the Public Health Service Policy on Humane Care and Use of Laboratory Animals, and the Office of Laboratory Animal Welfare, this study did not require review and approval. All rhino fecal samples were collected non-invasively from the ground during routine animal husbandry procedures. No animal activities were involved.

### Sample Collections

The targeted sample size for this study was six fecal samples from each rhinoceros species, and within each species, a minimum of three males and three females were to be included, except Sumatran rhinoceroses since only three individuals were available in the United States. All five collaborating facilities maintained their rhinos under intensively managed conditions during the sampling period. Animal care staff provided the daily diets and water, visually examined each rhino for health and/or injury, cleaned the enclosures and monitored environmental conditions both indoors and outdoors, giving rhinoceroses access to these habitats according to standard husbandry protocols. Staff at the holding facilities were asked to sample from fresh fecal boluses that were either found in individual rhino enclosures during the morning cleaning routine or collected shortly after an individual rhino was observed defecating. Boluses were broken open and sampled from the middle using a sterile scoop to avoid any environmental contamination. Fecal samples were only to be collected from clinically healthy rhinoceroses that were not being treated with any medication.

Fecal samples were collected from black rhinoceroses (*n* = 6; three female and three male), white rhinoceroses (*n* = 13; eight female and five male), GOH rhinoceroses (*n* = 9; five female and four male), and Sumatran rhinoceroses (*n* = 3; two male and one female). Rhinoceros ages ranged from 2.5 to 39.5 years. Samples were stored frozen (−20°C) and sliced into two aliquots each exceeding 0.5 g in weight. One set of samples was processed for 16S rRNA gene sequencing and the other set of matched samples was dried by lyophilization prior to metabolomic analysis. Two samples, one each from a GOH rhinoceros and white rhinoceros, were omitted from the final analyses of the 16S rRNA sequencing data due to a high proportion of soil bacteria reads suggesting excessive soil contamination in the sample and failed amplification attempts, respectively. The final list of samples included in the statistical analyses is shown in Table 1.

**TABLE 1:** Details about the rhinoceroses involved in the study and the general diet composition fed during the sample collection period.

**Species^a^**	**Iron overload disorder status**	**Facility**	**Age (years)**	**Sex**	**Month sampled**	**Diet (%)**			
						**Hay^b^**	**Pellet^c^**	**Browse**	**Produce**
BR	Susceptible	1	4	F	November	55	33	0^∗^	12
BR	Susceptible	3	18.5	M	February	76	19	0^∗^	5
BR	Susceptible	2	27	F	December	55	32	0^∗^	13
BR	Susceptible	5	16.5	F	October	40	41	18	0^∗^
BR	Susceptible	5	16.5	M	October	46	36	18	0^∗^
BR	Susceptible	5	19.5	M	October	40	41	18	0^∗^
GOH	Resistant	1	8	F	August	50	42	0^∗^	8
GOH	Resistant	1	21.5	F	August	46	39	0^∗^	15
GOH	Resistant	4	9	M	January	81	19	0	0^∗^
GOH	Resistant	4	10.5	M	December	81	19	0	0^∗^
GOH	Resistant	4	18	F	December	81	19	0	0^∗^
GOH	Resistant	5	16	F	October	67	33	0	0
GOH	Resistant	5	17	F	October	68	32	0	0
GOH	Resistant	5	39.5	M	October	65	35	0	0^∗^
SR	Susceptible	1	6	M	August	4	0	87	9
SR	Susceptible	1	9	F	February	5	0	84	11
SR	Susceptible	1	33	M	January	4	0	87	9
WR	Resistant	4	2	M	December	81	19	0	0^∗^
WR	Resistant	4	2.5	M	December	81	19	0	0^∗^
WR	Resistant	4	7	F	December	81	19	0	0^∗^
WR	Resistant	4	7	F	December	81	19	0	0^∗^
WR	Resistant	5	2.5	M	October	73	27	0	0
WR	Resistant	5	2.5	M	October	73	27	0	0
WR	Resistant	5	3.5	F	October	79	21	0	0
WR	Resistant	5	4	F	October	73	27	0	0
WR	Resistant	5	9.5	F	October	71	29	0	0
WR	Resistant	5	12	F	October	71	29	0	0
WR	Resistant	5	23.5	M	October	79	21	0	0
WR	Resistant	5	25	F	October	71	29	0	0

*^*a*^BR, black rhinoceros; GOH, greater one-horned rhinoceros; SR, Sumatran rhinoceros; WR, white rhinoceros; ^*b*^Hay types included: grass, timothy, peanut, coastal, alfalfa, and orchard grass. Often a mix of hays was fed; ^*c*^Pellet types included: Mazuri ADF-25, Mazuri ADF-16, Mazuri Moose Breeder, and specially formulated pellet; ^∗^Offered opportunistically or during operant conditioning training but not considered a staple of the daily diet.*

### DNA Extraction

Epicentre Catch-All foam swabs (Illumina, San Francisco, CA, United States) were moistened in sterile water and used to swab aliquots of each fecal sample until the swab tips were visibly stained with feces, yet free of excess debris (such as plant material in the feces). Swab tips were immediately placed into 2-ml screwcap vials (Sarstedt, Numbrecht, Germany) for DNA extraction. All specimens were extracted in one batch using the QIAamp DNA mini kit (Qiagen, Valencia, CA, United States; tissue protocol), according to instructions by the manufacturer, and eluted in 200 μl AE buffer. A tube without fecal material was processed in parallel to serve as a negative extraction control.

### PCR Amplification

The V4 hypervariable region of the 16S rRNA gene was amplified by PCR using barcoded universal fusion primers 515 forward (5′-GTGYCAGCMGCCGCGGTAA-3′) and 806 reverse (5′-GGACTACNVGGGTWTCTAAT-3′). The 25-μL PCR reactions were carried out in 96-well plates and run in triplicate using 0.4 μM concentrations of each commercially synthesized primer, 2.5× Hot MasterMix (5 PRIME) and 1 μL of each DNA template. As a negative control, a PCR reaction was also carried out in parallel without DNA template. Thermal cycling conditions consisted of an initial denaturing step of 94°C for 2 min, followed by 30 cycles of 95°C for 30 s, 53°C for 30 s, and 65°C for 30 s, with a final extension step of 65°C for 8 min. Triplicates were pooled and purified using the UltraClean 96 PCR Cleanup Kit per the manufacturer’s protocol (Polz and Cavanaugh, 1998). The Quant-iT High-Sensitivity dsDNA Assay Kit (Invitrogen) was then used to quantify the DNA concentrations of each well (mean = 7.47 ng/ul, range = 0.02–12.7 ng/ul). The amplicons were then pooled in a single tube at equimolar ratios using a liquid handler. This amplicon pool was concentrated by ethanol precipitation, resuspended in 100 uL of molecular biology-grade water (Life Technologies) and gel purified and recovered using a QIAquick Gel Extraction Kit (Qiagen). The Quant-iT PicoGreen dsDNA Assay Kit (Invitrogen) and a Tapestation 2000 instrument (Agilent) were used to quantify the final DNA concentration (16 ng/uL) of the purified amplicon pool. The Agilent Bioanalyzer was used to determine the average size of the amplicon library (389 bp). PCR products and negative controls were sequenced (paired end sequencing, 2 × 250 nucleotides) on a HiSeq 2500 instrument (Illumina) at the University of Illinois, Urbana–Champaign, IL, United States.

### 16S rRNA Sequence Read Processing

Primer sequences were removed using cutadapt v1.16 (Martin, 2011). Read processing and error correction was performed using the open-source R package DADA2 v1.8 (Callahan et al., 2016a). Quality filtering, error modeling, dereplication, denoising, and merging of paired end reads were conducted using the default parameters. Truncation of forward and reverse reads was performed at 240 and 220 nucleotides, respectively based on visual inspection of the Illumina quality scores. Reads were removed if an Illumina quality score was less than or equal to two, the maximum expected error rate for the forward or reverse read exceeded two, or the forward or reverse read contained an ambiguous base. The sample inference function (dada) was run using the default specification and each sample processed independently precluding the calling of singleton ASVs. Forward and reverse reads were merged after error correction to form an amplicon sequence variant (ASV) table. Chimera removal was implemented using the consensus method provided by the DADA2 function removeBimeraDenovo. Taxonomic classification was performed using the DADA2 formatted RDP v16 reference files. The assignSpecies and assignTaxonomy functions were used to combine exact genus- and species-level matching with an R implementation of the RDP Naive Bayesian Classifier to facilitate optimal assignment. Sequence alignment was performed using the default settings of the AlignSeqs function in the DECIPHER package v2.8.1 (Wright, 2016). A *de novo* phylogenetic tree was generated using the phanghorn package v2.4 (Schliep, 2011) to first construct a neighbor-joining tree and subsequent maximum likelihood tree with the neighbor-joining tree as the starting point as recommended by Callahan et al. (2016b). Outgroup rooting was accomplished by selecting the longest individual branch. Phyloseq v1.24 (McMurdie and Holmes, 2013) was used to integrate the sample metadata, ASV table, phylogenetic tree, and taxonomic assignments for statistical analyses. A total of 65 and 111 reads and nine ASVs were retained in the negative control samples, respectively. Four of these ASVs were also observed in rhino specimens including an abundant ASV (ASV1) which was classified to the genus *Ruminococcus*. The other three ASVs were all Proteobacteria and classified as *Escherichia* (ASV2832), *Acinetobacter* (ASV3207), and *Vampirovibrio* (3986) organisms and were detected in ten, four, and six rhino samples, respectively. All four ASVs were retained in downstream analyses as we could not rule them out as true members of the rhino microbiota.

### Sample Preparation for Metabolomics Analyses

All dried fecal test samples were weighed (95–162 mg) and transferred into 2 mL tubes containing 2.8 mm ceramic beads (VWR) prior to processing. On the day of processing, 1.5 mL of cold PBS was added to each tube and the samples were homogenized for 30 s at 5000 rpm using Minilys (Bertin Technologies) Samples were then transferred into 2 mL tubes without beads and centrifuged at 10,000 × *g*_*n*_ at 4°C for 20 min. Supernatants were filtered at 12,000 × *g*_*n*_ for 60 min at 4^°^C using pre-washed 3 kDa spin filters (NANOSEP 3K, Pall Life Sciences). The nuclear magnetic resonance (NMR) buffer containing 100 mM phosphate buffer in D_2_O, pH 7.3, and 1.0 mM TMSP (3-Trimethylsilyl 2,2,3,3-d_4_ propionate) was added to 540 uL of fecal filtrate. The final sample volume was 600 uL and final TMSP concentration in each sample was 0.1 mM.

### NMR Spectroscopy Data

The experiments were conducted using 550 μL samples in 103.5 mm × 5 mm NMR tubes (Bruker Analytik, Rheinstetten, Germany). One-dimensional ^1^H-NOESY NMR spectra were acquired on a Bruker Avance II 600 MHz spectrometer. All data were collected at a calibrated temperature of 298K using the noesygppr1d pulse sequence in the Bruker pulse sequence library. Experiments were run with 4 dummy scans and 256 acquisition scans with an acquisition time of 3.4 s and a relaxation delay of 3.0 s. The NOESY mixing time was 6 ms. The spectral width was 12 ppm, and 64K real data points were collected. All free induction decays were subjected to an exponential line-broadening of 0.3 Hz. Upon Fourier transformation, each spectrum was manually phased, baseline corrected, and referenced to the internal standard TMSP at 0.0 ppm using Topspin 3.5 software (Bruker). Two-dimensional data, ^1^H–^1^H total correlation spectroscopy (TOCSY) and ^1^H–^13^C heteronuclear single quantum coherence (HSQC), were collected for metabolite assignment on representative samples.

### Metabolomics Data Analysis

Metabolites were assigned by comparing the chemical shifts with reference spectra found in databases, such as the Human Metabolome Database (Wishart et al., 2018), and Chenomx^®^ NMR Suite profiling software (Chenomx Inc., version 8.1). A total of 36 metabolites were assigned and quantified using Chenomx software based on the internal standards. Prior to statistical analysis, normalization by dry sample weights and mean-centered scaling were applied.

### Statistical Analysis

Means with standard deviations, frequencies, and percentages were used to describe the rhinoceros characteristics and habitats. Proportions were used to quantify the fraction of total reads mapped to each taxonomic level from phylum to species. Heat trees as generated by the metacoder v0.3.2 package (Foster et al., 2017) were used to visualize the number of reads mapping to taxonomic levels from kingdom to genus. Stacked bar charts were used to describe the relative abundance of major bacterial phyla for each rhinoceros species. Kruskal–Wallis or Wilcoxon rank-sum tests were used to test for differences in Shannon diversity according to rhinoceros species, sex, facility and hemosiderosis risk. Spearman correlations were used to assess the correlation between Shannon diversity and age. Principal components analysis (PCA) was conducted on the raw counts after variance stabilizing transformation as implemented in DESeq2 v1.24 (Love et al., 2014). PCA was also conducted on the raw counts after a centered log-ratio transformation (Aitchison distance) and principal coordinates analysis (PCoA) performed on the Bray–Curtis dissimilarity matrix and weighted and unweighted UniFrac distances after subsampling to the lowest observed read depth (45,074 reads) to assess the robustness of the beta-diversity ordinations to choices in the normalization procedure and distance measure. Differences in beta-diversity were tested by permutational multivariate analysis of variance (PERMANOVA) as implemented by the Adonis function in vegan v2.5.5 (Oksanen et al., 2019) and differences in beta dispersion tested using the betadisper and permutest functions at the default settings. Differentially abundant ASVs in IOD-susceptible vs. IOD-resistant rhinoceros species were identified using moderated negative binomial regression as implemented by DESeq2 after filtering to include ASVs detected in at least half of all samples. Facility was included as a covariate in all models and ASVs with a Benjamini–Hochberg false discovery rate (FDR) corrected *p*-value of <0.1 reported. Wilcoxon rank-sum tests were performed on the subsampled taxa as a sensitivity analysis for differential abundance.

Principal components analysis was performed on the normalized metabolite concentrations to assess the clustering of stool specimens based on rhinoceros facility or species. PERMANOVA was used to test for differences in the overall metabolite concentrations. Linear regression was used to test for differences in metabolite concentrations between IOD-susceptible and IOD-resistant rhinoceros species. Rhinoceros facility was included as a covariate and metabolites with a FDR *p*-value of <0.1 reported. Correlations between bacterial families and fecal metabolites according to IOD susceptibility status and within the black rhinoceros species were visualized using heatmaps. ASVs were aggregated to the family level and the top 30 families seen in at least three samples in both IOD-susceptible and black rhinoceros species selected. Heatmaps were generated using the ComplexHeatmap v2.0.0 package (Gu, 2016). All analyses were conducted using R v3.6.0 (R Core Team, 2019) including the tidyverse (version: 1.2.1; Wickham, 2017), phyloseq (version: 1.28.0; McMurdie and Holmes, 2013), vegan (version: 2.5.5; Oksanen et al., 2019), microbiome (version: 1.6.0; Lahti, 2018), DESeq2 (version: 1.24.0; Love et al., 2014), ComplexHeatmap (version: 2.0.0; Gu, 2016), circlize (version: 0.4.6; Gu, 2014), Metacoder (version: 0.3.2; Foster et al., 2017), and Cowplot (version: 1.0.0; Wilke, 2019) packages.

### Data Deposition

The 16S rRNA data sets generated in this study were deposited in the NCBI BioProject database under accession number PRJNA558964.

## Results

### Microbiota of IOD-Susceptible Rhinoceros Species Cluster Together and Are Less Diverse Than Those of IOD-Resistant Rhinoceros Species

**FIGURE 2:**
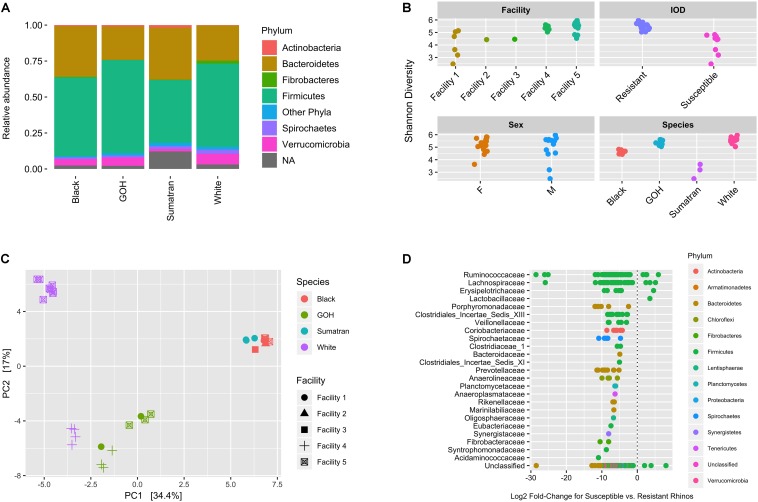
Microbial community composition, diversity, and differential abundance. **(A)** Fecal community relative abundance at the phylum level according to rhino species. **(B)** Shannon diversity according to facility, IOD risk, sex, and species. **(C)** Principal coordinates analysis performed on the Bray–Curtis dissimilarity metric. **(D)** Differentially abundant core ASVs in IOD susceptible vs. resistant rhinos. Log2 fold-changes shown for ASVs with a BH-FDR corrected *p*-value < 0.1 (356 of 446 core taxa). Each dot reflects a distinct ASV. ASVs are presented by bacterial family on the *y*-axis and colored by phylum. ASVs with log2 fold-change values greater than zero are more abundant in IOD-susceptible rhino species. ASVs with log2 fold-change values less than zero are more abundant in IOD-resistant rhino species.

A total of 2,303 ASVs, representing 18 phyla, were detected across all rhinoceros species. Firmicutes represented the highest relative abundance (range; 66.3–51.0%) followed by Bacteroidetes (39.8–23.4%) (Figure 2A). Also present in all species, but at much lower abundance, were Verrucomicrobia (7.6–1.9%), Spirochetes (3.1–1.1%), Actinobacteria (1.04–0.03%), and Fibrobacteres (2.14–0.19%). The mean log-ratio of Firmicutes to Bacteroidetes was 0.41 ± 0.61 for IOD-susceptible and 0.98 ± 0.47 for IOD-resistant species (*p* = 0.011). Although 97–98% of all reads were successfully mapped to a bacterial phylum for white, black, and GOH rhinoceroses, only 88% of Sumatran rhinoceros reads were mapped to a phyla (Supplementary Table 1A) due to a few unidentified ASVs with high read counts. Subsequent BLAST analysis against the NCBI database showed an abundant ASV had >90% coverage and 80% identity to numerous *Clostridium* sequences, whereas another abundant ASV was found to have <80% identity to all alignments. In general, however, the majority of ASVs mapped to the family level and above were found across rhinoceros species (Supplementary Table 1B), including those affiliated with the orders Bacteroidales and Clostridiales and the Families Ruminococcaceae, Spirochaetaceae, and Coriobacteriaceae. Heat trees showing the number of ASV mapped to taxonomic ranks are provided as Supplementary Figure 1. Despite the higher level taxonomic similarities in microbial orders and families, the presence of specific ASVs differed across rhinoceros species. Supplementary Figure 2 highlights differences in the presence/absence of ASVs for selected subsections in many parts the phylogenic tree. For example, many ASVs mapping to Coriobacteriaceae, Prevotellaceae, and Clostridium XlVa were not detected in IOD-susceptible rhinos.

Shannon diversity within rhinoceros fecal samples differed according to facility (*p* = 0.007), rhinoceros species (*p* < 0.001) and IOD susceptibility (*p* < 0.001; Figure 2B). Age was inversely associated with Shannon diversity (Spearman rho = −0.45, *p* = 0.015), but no difference was observed for sex (*p* = 0.914). PCoA performed on the Bray–Curtis dissimilarity matrix indicated clustering of samples based on rhinoceros species and facility, and IOD-susceptibility (Figure 2C). Rhinoceros species explained the greatest amount of variance (*R*^2^ = 0.49; *p* < 0.001) followed by facility (*R*^2^ = 0.17; *p* < 0.001; Supplementary Table 2), whereas age and sex were not significant (*R*^2^ = 0.02; *p* = 0.253 and *R*^2^ = 0.01; *p* = 0.488, respectively). Ordinations performed after variance stabilizing or centered log-ratio (i.e., Aitchison distance) transformation produced similar clustering (Supplementary Figure 3). Clustering based on weighted and unweighted UniFrac distances was less pronounced for IOD-susceptible, and more pronounced for IOD-resistant species, suggesting importance for the relative abundances of microbial taxa in these rhinoceros species. However, samples from GOH and White rhinoceroses generally separated from those collected from Sumatran and Black rhinoceroses along the first axis regardless of approach.

A large proportion (356/446) of abundant ASVs differed (with FDR *p*-values < 0.1) between IOD-susceptible and -resistant groups (Figure 2D). Almost all of these ASVs were more abundant in IOD-resistant rhinoceroses, consistent with the greater Shannon diversity in these species. In fact, with the exception of a few ASVs mapping to Firmicutes, many of these ASVs were not consistently detected or were much less abundant in IOD-susceptible rhinos. Similar results were obtained with the Wilcoxon rank-sum test (Supplementary Table 3) performed on the subsampled data (392/446 abundant ASVs with FDR *p*-value < 0.1) and when testing for differential abundance according to rhino species via DESeq2 (441/446 abundant ASVs with FDR *p*-value < 0.1; 81% of these ASVs overlapped with those detected for IOD status; Supplementary Table 4).

### The Black Rhinoceros Metabolome Is Distinct Among the Rhinoceros Species Containing Higher SCFA Concentrations

**FIGURE 3:**
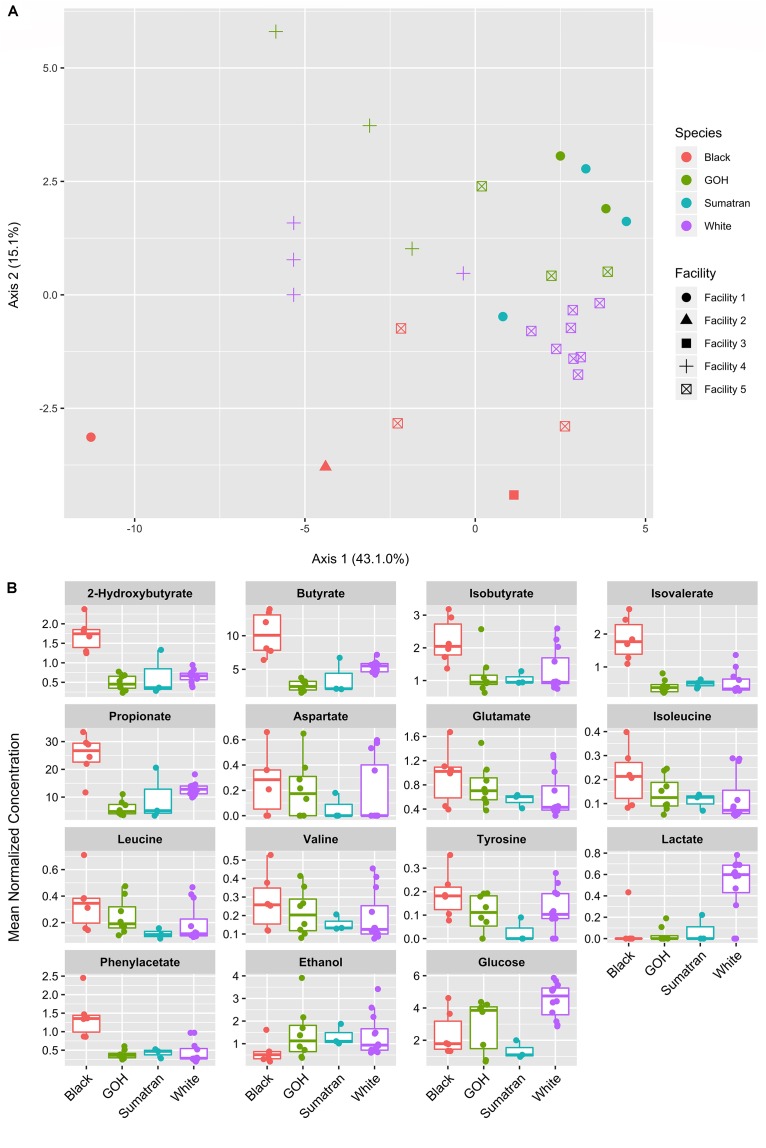
Fecal metabolite ordination and differential abundance. **(A)** Principal components analysis of 36 fecal metabolites. **(B)** Boxplots of differentially abundant fecal metabolites (15 of 36) according to rhino species. Plots shown where the FDR corrected *p*-value < 0.1 for IOD-susceptible vs. IOD-resistant rhinos.

A total of 36 polar metabolites were identified and quantified in the fecal samples. PCoA was used to project the relative relationships of overall metabolic profiles among samples from each of the species and facilities (Figure 3A). Both factors significantly affected the metabolic profiles (PERMANOVA *p*-values < 0.001). In contrast, rhinoceros age and sex were not significant (*p* = 0.634 and 0.332, respectively). A total of 15 out of 36 analyzed metabolites differed in abundance (with FDR corrected *p*-value < 0.1) between susceptible and resistant groups. These metabolites were further analyzed among host species and the results presented in boxplot format (Figure 3B) to better identify how the variance was distributed among and within the species. The black rhinoceros stood out as the primary source of variation with a higher abundance of SCFAs (2-hydroxybutyrate, butyrate, isobutyrate, isovalerate, and propionate) compared to the other three species. In general, samples from the Sumatran rhinoceroses fell in line with those from the GOH and white rhinoceroses, but were relatively impoverished for many amino acids (aspartate, glutamate, isoleucine, valine, and tyrosine). With the exception of two individuals, white rhinoceroses were unique in exhibiting a high abundance of lactate compared to the other rhinoceros species. Interestingly, the white rhinoceros data for isobutyrate and some of the amino acids (aspartate, glutamate, valine, and tyrosine) formed high and low concentration groups based on their facility origin (Facility 4 = high concentration; Facility 5 = low concentration). These metabolites were likely strong drivers of the significant facility effect in the statistical model as shown in PCoA scores plot.

**FIGURE 4:**
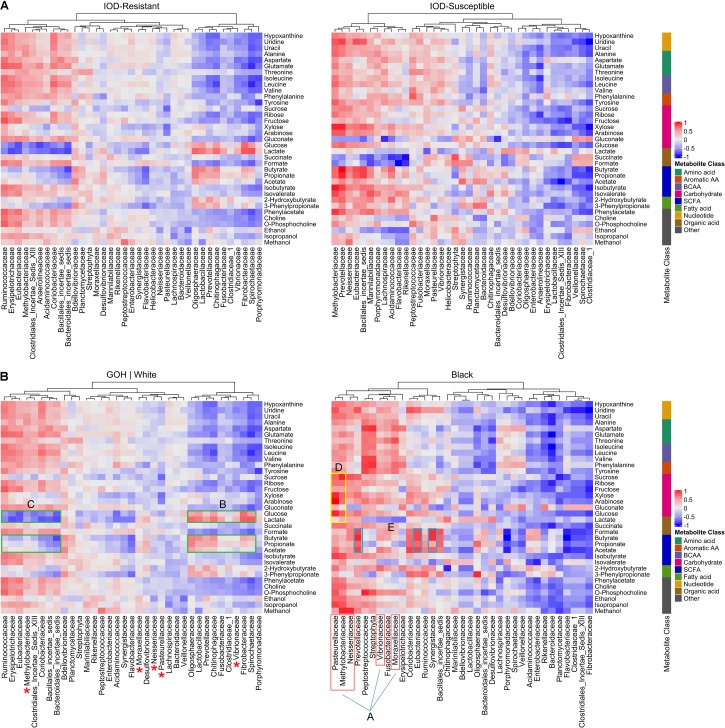
Heatmap showing the Spearman correlation between fecal bacterial families and metabolites according to IOD susceptibility status **(A)**. Heatmaps exhibiting the Spearman correlation between fecal bacterial families and metabolites in IOD-susceptible black rhinoceroses and the IOD-resistant white rhinoceroses and GOH rhinoceroses **(B)**. A-red, proteobacteria in black rhinoceroses positively correlated with most metabolites. Red asterisks indicate proteobacteria families demonstrating little to no correlation with any metabolites in IOD-resistant rhinoceroses. In IOD-resistant species: B-green, bacterial families positively correlated with glycolytic metabolites and SCFAs; C-green, families negatively correlated with sugars and SCFAs. In black rhinoceroses: D-yellow, proteobacteria exhibiting strong positive correlations with sugars; E-blue, bacterial families exhibiting the highest correlations to SCFAs.

Heatmaps displaying the Spearman correlations between fecal bacterial families and metabolites were initially developed for IOD-resistant and IOD-susceptible species separately, and correlation pattern differences were noted between these two groups (Figure 4A). However, the metabolome signature of the black rhinoceros was distinct among the four species, and combining results from the few Sumatran rhinoceros samples with those of the black rhinoceros samples obscured the extent to which black rhinoceroses differed from their counterparts. Therefore, heatmaps were produced to demonstrate the differences between black rhinoceros and the white/GOH rhinoceroses (IOD-resistant group; Figure 4B).

The differences in correlations between microbial families and metabolite abundances strongly suggested that different microbial populations influenced the gut metabolomic environment in each of the rhinoceros groups. For example, several families belonging to the Proteobacteria phylum, such as Vibrionaceae, Moraxellaceae, and Neisseriaceae were positively correlated with many of the metabolites detected in black rhinoceroses (A, red box). Furthermore, Pasteurellaceae and Methylobacteriaceae exhibited strong positive correlations with all sugar metabolites (e.g., glucose, xylose, and arabinose; D, yellow box). The families exhibiting the highest correlations to SCFAs included Prevotellaceae, Eubacteriaceae, and Synergistaceae, well-known SCFA producers (Ríos-Covián et al., 2016; E, blue boxes). In contrast, many of these Proteobacteria families demonstrated little to no correlation with any metabolites in IOD-resistant rhinoceroses (red ^∗^).

In IOD-resistant species, strong correlation patterns revealed links between the bacterial families and metabolic processes. The bacterial families positively correlated with glycolytic metabolites (glucose and lactate), such as Bacteroidetes, Spirochaetaceae, and Fibrobacteraceae, also demonstrated positive correlations with microbial metabolites such as the SCFAs (B, green boxes). In contrast, members of Clostridiales and Firmicutes were negatively correlated with both sugars and SCFAs (C, green boxes).

## Discussion

In this study, the gut microbiomes and metabolomes among four of the five extant rhinoceros species were characterized and compared, and an association was identified between gut microbiome and IOD susceptibility. Within the two distantly related, IOD-susceptible rhinoceros species, our findings demonstrated shared features of the distal gut microbiota, differences with their two IOD-resistant counterparts and divergence in metabolite profiles.

Previous large wildlife studies that included a few rhinoceros samples demonstrated that host phylogeny and diet influenced bacterial diversity, and the most abundant phyla in most mammals were Firmicutes and Bacteroidetes (Ley et al., 2008; Youngblut et al., 2019). Therefore, the dominance of these two phyla members in the gut microbiota of rhinoceroses across several North American zoos was expected and in accordance with reports of rhinoceros microbiome in China (Bian et al., 2013), Europe (Antwis et al., 2019), San Diego (Williams et al., 2019), South Africa and Texas (Gibson et al., 2019).

The Sumatran rhinoceros is the closest living relative of the wooly rhinoceros (Willerslev et al., 2009), but the analysis of a single wooly rhinoceros gut revealed very small numbers of Bacteroidetes (0.2% of reads) with Firmicutes dominating at 68% followed by Proteobacteria (19.2%) (Mardanov et al., 2012). The latter was not identified in appreciable abundance in the Sumatran rhinoceros. In contrast, Proteobacteria were more abundant in female black rhinoceroses in European zoos, particularly in two post-partem samples (Antwis et al., 2019) and in wild black rhinoceroses in South Africa (Gibson et al., 2019).

Interestingly, the two IOD-susceptible species, the Sumatran and black rhinoceroses, contained higher proportions of Bacteroidetes than the IOD-resistant GOH and white rhinoceroses. Similarly, Ley et al. (2008) reported a higher proportion of Bacteroidetes in the black rhinoceros compared to the GOH rhinoceros. Previous studies have reported a shift favoring Bacteroidetes in the Firmicutes:Bacteroidetes ratio associated with several factors including colitis (Costa et al., 2012), increased dietary fiber (De Filippo et al., 2010), and obesity (Larsen et al., 2010; Schwiertz et al., 2010). However, results are mixed regarding the latter (Ley et al., 2006; Guo et al., 2008; Turnbaugh et al., 2008; Turnbaugh and Gordon, 2009; Sze and Schloss, 2016) suggesting no strong association exists. Bacteroidetes are known for producing enzymes that degrade dietary polymers such as plant cell wall compounds which otherwise would pass through the gut undigested (Thomas et al., 2011). Given the tree branches, bark, bushes and shrubs preferred by browsers over more protein-rich grasses consumed by grazers, a shift toward greater Bacteroidetes populations may reflect the mutually beneficial relationship between the host and its gut microbiota that evolved over time.

There have been many reports of a shift in microbial populations with pathogenic microbes increasing in the presence of iron or iron supplementation (Zimmermann et al., 2010; Jaeggi et al., 2015; Manzo and Bhatt, 2015; La Carpia et al., 2016). If the same occurs in rhinoceroses susceptible to IOD in response to elevated body iron load, it could help substantiate the suspected link between IOD and increased disease susceptibility in the black rhinoceros. Unfortunately, the inability to classify a high proportion of reads to the species, genus or even family level, precluded that goal. Unclassified sequences at deeper taxonomic levels are common with 16S rRNA sequencing due to the inability to discriminate between closely related taxa over the amplicon spanning region and incomplete reference databases. Most gut microbiome research has focused on rodents and humans, but microbial populations in wildlife, and even horses, are largely uncharacterized much beyond the phyla level (Daly et al., 2001; Ley et al., 2008; Proudman et al., 2015), with the exception of a recent study (Youngblut et al., 2019). A previous paper reported 15–42 and 58–85% unclassified bacteria at the family and genus levels, respectively, across five white rhinoceroses (Bian et al., 2013), and more recent publications on rhinoceros microbiota also reported high proportions of unmapped reads (Gibson et al., 2019; Williams et al., 2019). Similarly, we report 26–52 and 62–78% unclassified reads within family and genus, respectively, across all four rhinoceros species. Therefore, it could be misleading to compare mapped microbial populations among rhinoceros species at deeper taxonomic levels since a high proportion of unclassified reads may not map to available reference sequences.

Our data demonstrated clustering of microbiotas based on host IOD-susceptibility. Despite phylogeny being one of the main drivers of gut microbiome (Ley et al., 2008; Youngblut et al., 2019), the white rhinoceros and black rhinoceros microbiotas were not closely associated. Instead, the IOD-susceptible Sumatran rhinoceros and black rhinoceros clustered together even though they are more distantly related with non-overlapping historic distributions on two different continents (Africa and Asia^1^). Additionally, the two IOD-resistant, grazing GOH and white rhinoceros species were more closely aligned with each other than with their respective continental counterparts. Furthermore, gut microbiota diversity is desirable for overall organismal health (Le Chatelier et al., 2013), and the Sumatran rhinoceros and black rhinoceros harbored less microbial diversity than the GOH rhinoceros and white rhinoceros. Considering that the former are browsers that habitually consume a more diversified, high fiber diet than their grazing cousins, the opposite result was anticipated. Although black rhinoceroses and Sumatran rhinoceroses are strict browsers in the wild, in a zoo setting, black rhinoceroses do consume a high proportion of hay in their daily diet, much like their grazing counterparts, whereas the captive Sumatran rhinoceros’s diet primarily consists of browse (Table 1; Dierenfeld et al., 2000). Regardless of these significant dietary differences between zoo-maintained black rhinoceroses and Sumatran rhinoceroses, their microbiota profiles suggested that something other than phylogeny and diet influences gut microbial communities within the Rhinocerotidae family. Surprisingly, a study comparing the microbiotas of wild and captive wildlife reported increased alpha diversity in zoo-managed black rhinoceroses and white rhinoceroses compared to their wild counterparts (McKenzie et al., 2017). However, the difference may be explained by location and uneven sampling size. Wild samples were collected from rhinos in a single reserve and only included one black rhinoceros, whereas captive rhino samples were from a mix of United States and European zoos and included six black rhinoceroses. In contrast, a recent study reported higher alpha diversity in wild black rhinoceroses in South Africa compared to black rhinoceroses maintained at two facilities in Texas (Gibson et al., 2019).

Although facility/location can impact the microbiota, we could not fully determine the independent contribution of facility to differences in alpha- or beta-diversity due to imbalanced species contribution across facilities and inconsistent findings. For example, Facility 4 did not contribute samples from any IOD-susceptible individuals. However, black rhinoceroses in our study came from four different facilities with varied diets (Table 1) and still clustered together with the Sumatran rhinoceroses. Similarly, the GOH rhinoceroses were housed at three facilities and they also clustered together. These data suggest that facility did not significantly influence the microbiota in these two rhinoceros species. In contrast, white rhinoceroses separated by location. Regardless, host species was the predominant factor influencing variation in microbiota structure, whereas neither age nor sex significantly affected clustering.

The gut microbiota contributes substantially to the fecal metabolome (Martin et al., 2007; Thomas et al., 2011; Flint et al., 2015), especially in herbivores that rely on their microbial populations to breakdown plant cell membranes and convert complex carbohydrates to more readily digestible compounds. Given the close relationship between the gut microbiotas of Sumatran rhinoceroses and black rhinoceroses, a similar relationship of their fecal metabolomes was expected. However, in this study, the black rhinoceros had a distinct fecal metabolome in comparison with the other three species. In contrast, the Sumatran rhinoceros metabolome was more closely associated with those of the GOH rhinoceros and white rhinoceros. Williams et al. (2019) also reported similar metabolite composition between GOH and white rhinoceroses despite differences in gut microbial composition.

Short chain fatty acids (acetate, isobutyrate, isovalerate, propionate, and butyrate) are microbial fermentation products of the large intestine (Cummings, 1984) and an important energy source for the host. Our data suggested that the black rhinoceros fecal metabolome has higher levels of SCFAs than the other three rhinoceros species. These findings are intriguing since SCFAs, especially butyrate and propionate, are implicated as important mediators of obesity and metabolic syndromes (reviewed by Ríos-Covián et al., 2016). Several studies have demonstrated that total fecal SCFA concentrations increase with obesity and its associated metabolic disturbances in humans, and decrease with anti-obesity treatment (Schwiertz et al., 2010; Patil et al., 2012; Fernandes et al., 2014; Rahat-Rozenbloom et al., 2014; Ríos-Covián et al., 2016). Schook et al. (2015) reported that black rhinoceroses in zoos exhibit elevated inflammatory markers (tumor necrosis factor alpha, serum amyloid A and insulin), similar to those documented in obese humans. They suggested that metabolic disturbance could be the primary disrupter of black rhinoceros health, and that iron storage may be a secondary response. Additionally, serum ferritin, an acute phase protein, is extraordinarily high in some black rhinoceroses that are not sick from IOD (Wojtusik and Roth, 2018). Hyperferritinemia occurs in obese humans in the presence or absence of excess body iron load, and it is now known that up to one third of non-alcoholic fatty liver disease (NAFLD) patients have elevated iron levels, a condition termed “dysmetabolic iron overload syndrome” (Aigner et al., 2015). SCFAs are essential for maintaining intestinal epithelium physiology by modulating the response to inflammatory/infectious stimuli (Corrêa-Oliveira et al., 2016). Given the previously reported evidence for unhealthy inflammatory and/or metabolic states in the black rhinoceros, it is possible the elevated fecal SCFAs reported in this study are a response to those conditions.

Despite its similarity with black rhinoceroses in gut microbiota taxa composition, the Sumatran rhinoceros produced SCFA concentrations similar to those of the GOH rhinoceros and white rhinoceros. Although both black rhinoceroses and Sumatran rhinoceroses are susceptible to IOD, Sumatran rhinoceroses have presented with classic hemochromatosis in the absence of other disease states, whereas black rhinoceroses typically die of another primary cause and hemosiderosis is noted post-mortem (Dennis et al., 2007; Olias et al., 2012; Paglia and Tsu, 2012). Furthermore, serum ferritin concentrations in healthy black rhinoceroses can be significantly higher than those measured in Sumatran rhinoceroses that die of hemochromatosis (Roth et al., 2017; Wojtusik and Roth, 2018), Not only do these observations and data reveal the inadequacy of serum ferritin as a biomarker for IOD progression in the rhinoceros, they also suggest that the iron overload condition of these two species may differ in origin, etiology and/or impact.

All but two white rhinoceroses exhibited significantly elevated lactate concentrations compared to the other three rhinoceros species. Gut lactate accumulation can be detrimental, leading to colitis in humans (Vernia et al., 1988; Hove et al., 1994); lactate levels have been used as a measure of colic severity in horses (Latson et al., 2010). Host intestinal absorption of butyrate is preferred to lactate, thus bacterial cross-feeding that reduces intestinal lactate and increases butyrate or propionate production can benefit the host (Flint et al., 2015). Given that gastrointestinal tract ailments are one of the top health concerns and causes of death in zoo-maintained white rhinoceroses (Roth et al., 2019) and are also strong indicators of poor adaptation to translocation procedures (Miller et al., 2016), further research is warranted in identifying differences among rhinoceroses in microbial populations that lead to lactate production and conversion.

Finally, fecal ethanol levels were significantly lower in the black rhinoceros compared to the other three species. An increase in ethanol production has been linked to obesity and NAFLD in humans and mice (Cope et al., 2000; Raman et al., 2013). Therefore, this finding does not support the hypothesis that black rhinoceroses are suffering from metabolic disturbances associated with obesity. However, “normal” fecal metabolite values for individual species are highly variable, so it is possible that ethanol is high compared to that in wild black rhinoceroses.

Whereas this study has many strengths, there are also limitations. First, the sample size and unequal distribution of rhinoceros species across facilities limited our ability to disentangle the effect of facility from rhinoceros species for several analyses. Second, the time of the year in which stool specimens were collected was not standardized. While we saw little systematic difference in microbial composition or diversity according to season (Supplementary Figure 4), we cannot rule out more modest effects.

In conclusion, we found intriguing differences in gut microbial community composition and diversity in IOD-susceptible vs. IOD-resistant rhinoceros species despite the phylogenetic disparity within each group. The IOD-susceptible rhinoceroses exhibited less microbial diversity, which could have health implications. These findings provide the first insight into a relationship that clearly warrants further investigation given the strong association between microbiome and IOD susceptibility in this study. Furthermore, the black rhinoceros metabolome was distinct among the four rhinoceros species, a finding congruent with evidence of metabolic disturbance in serum profiles reported in zoo-housed black rhinoceroses (Schook et al., 2015). Such metabolic changes could be associated with hemosiderosis and the high susceptibility to infectious disease not observed in any of the other rhinoceros species in human care.

## Data Availability Statement

The 16S rRNA data sets generated in this study were deposited in the NCBI BioProject database under accession number PRJNA558964.

## Author Contributions

TR conceived the study, acquired the samples, and drafted the manuscript. NO performed bioinformatic analyses, interpreted the data, produced figures, and contributed to the writing of the manuscript. MW-C performed all metabolomics experiments and contributed to the data interpretation, figure production, and writing of the manuscript. AS and EB conducted the microbiome experiments and contributed to the data interpretation and writing of the manuscript. DR contributed to the data interpretation, and writing and editing of the manuscript. LR-R contributed to the writing and editing of the manuscript.

## Conflict of Interest

The authors declare that the research was conducted in the absence of any commercial or financial relationships that could be construed as a potential conflict of interest.

## Footnotes

1 https://rhinos.org/
