# Supplementary material for: Reduced Gut Microbiome Diversity and Metabolome Differences in Rhinoceros Species at Risk for Iron Overload Disorder
Source: Front Microbiol. 2019 Oct 4;10:2291. doi: 10.3389/fmicb.2019.02291 (PMC6792462; doi:10.3389/fmicb.2019.02291)
Supplement: Supplementary file 1 [file Data_Sheet_1.pdf]

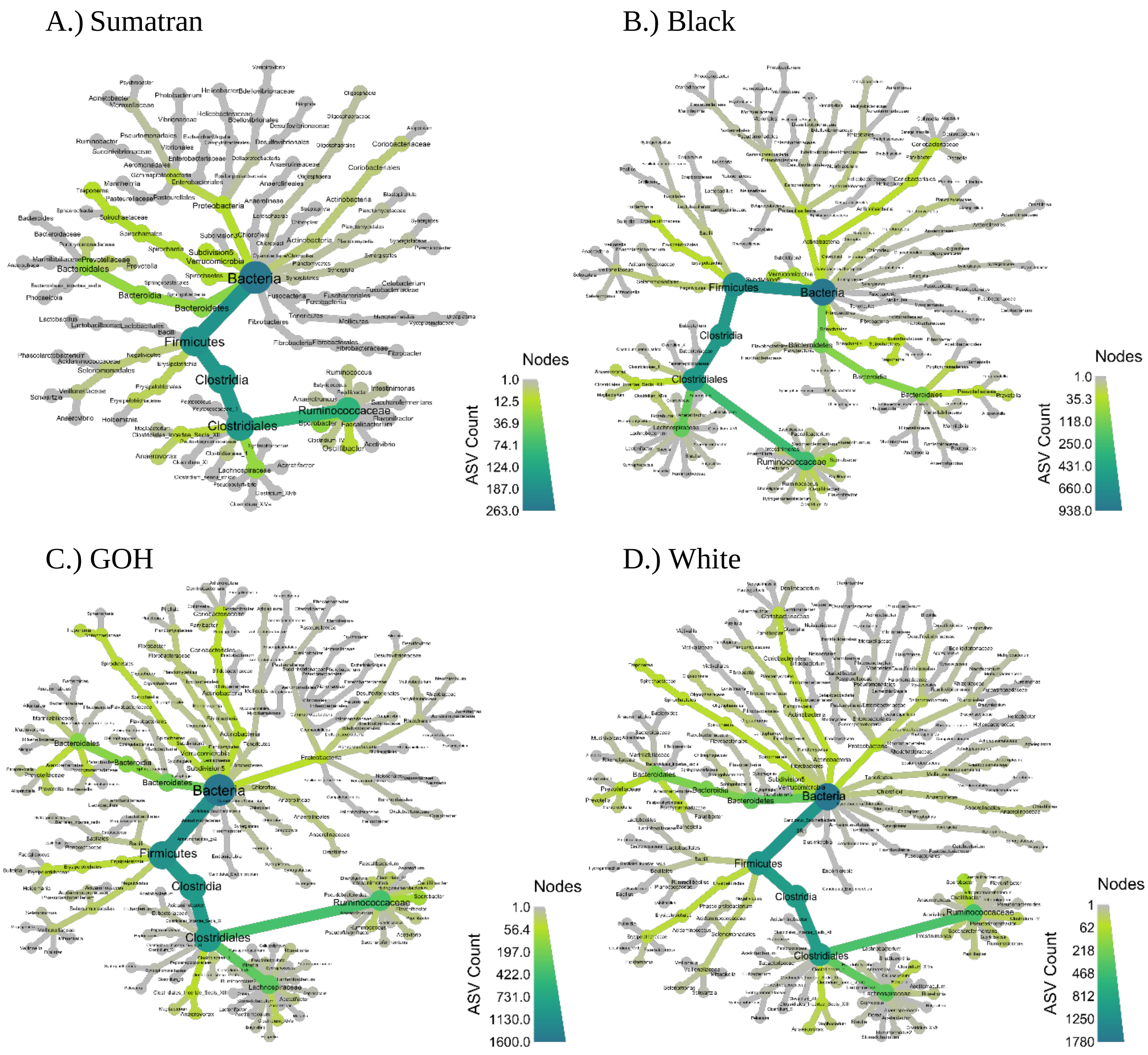

Supplemental Figure 1. Heat tree for each rhino species showing the number of ASVs mapped to the RDP version 16 training set using the assignTaxonomy and addSpecies functions provided in the R package dada2 version 1.8.0. Node and edges sized and colored according to the number of mapped ASVs.

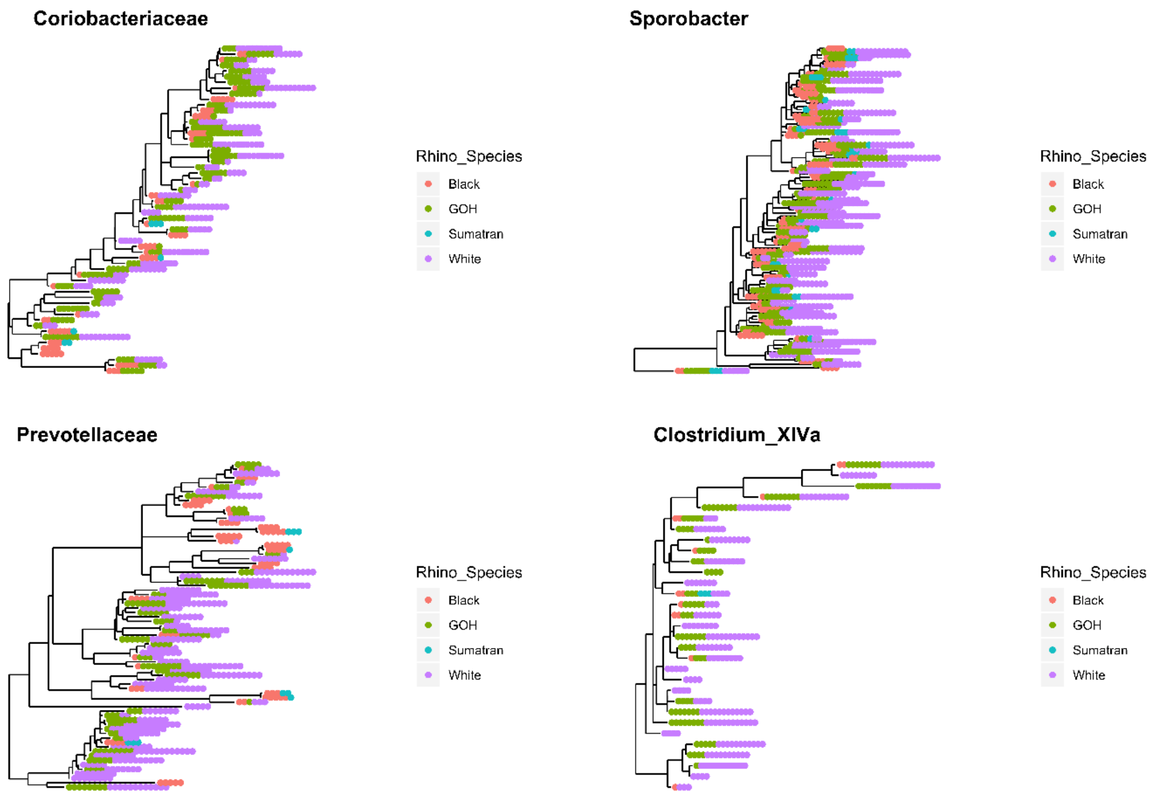

Supplemental Figure 2. Differences in ASV presence/absence in specific branches of the phylogenetic tree. Dots reflect the number of species in which an ASV was detected.

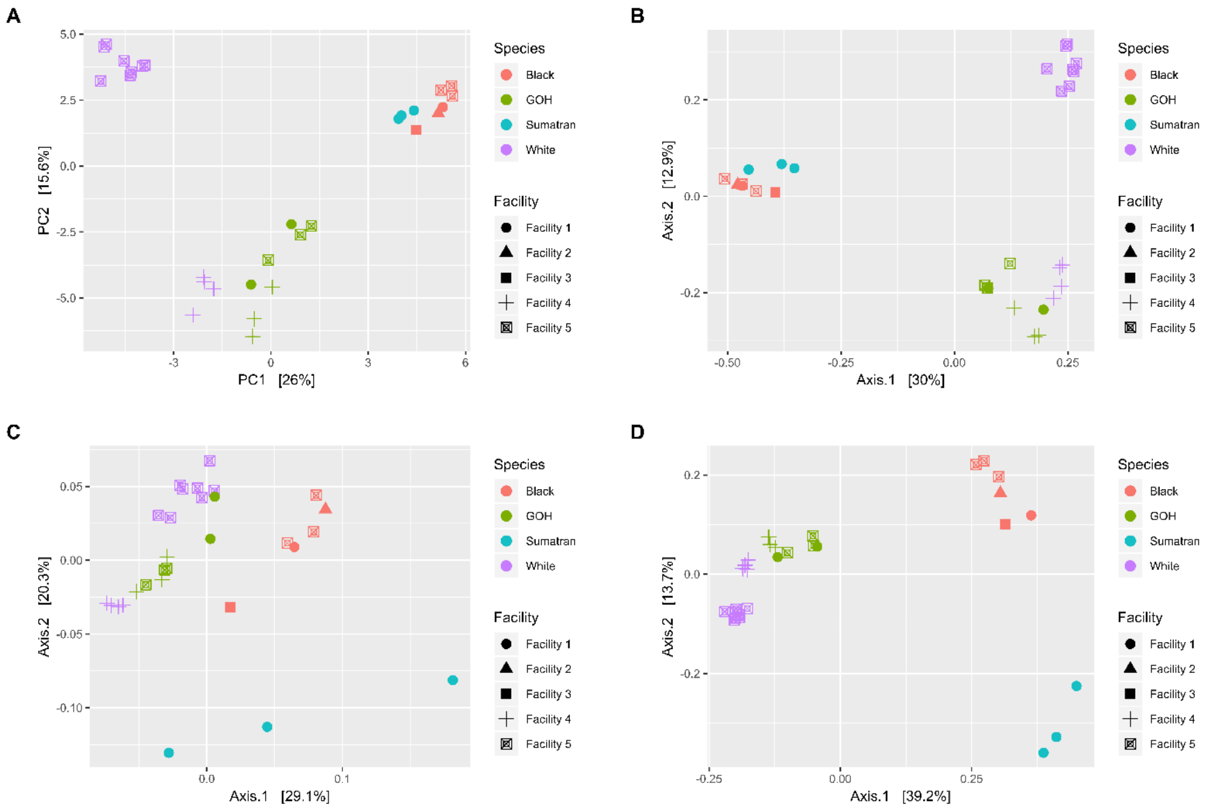

Supplemental Figure 3. Principal components or coordinates analysis of rhino fecal samples for selected distance matrices. **A.**) Centered log-ratio. **B.**) Bray-Curtis dissimilarity. **C.**) Weighted UniFrac. **D.**) Unweighted UniFrac.

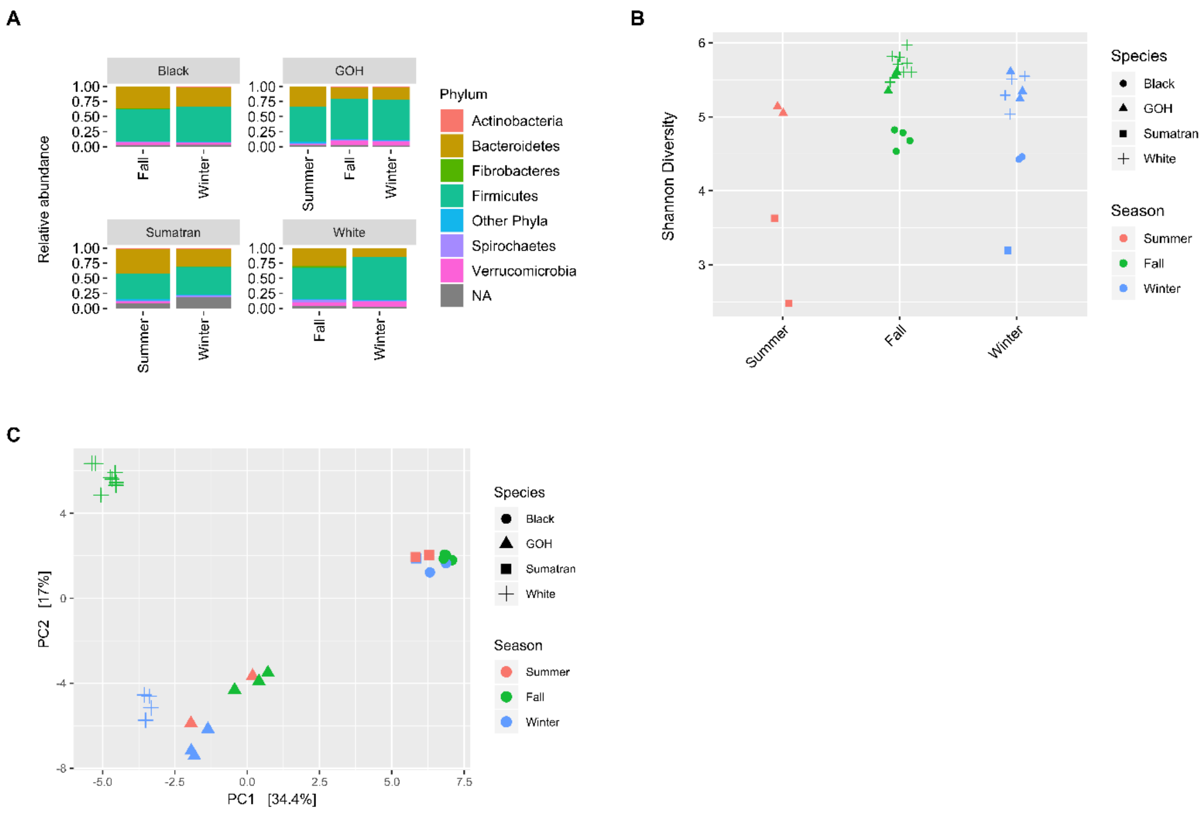

Supplemental Figure 4. Microbial community composition and diversity according to season of sample collection. **A.**) Fecal microbiota phylum-level relative abundance according to season and rhino species. **B.**) Shannon diversity according to season and rhino species. **C.**) Principal components analysis performed on raw counts after variance stabilizing transformation as implemented in DESeq2 version 1.24.0.

Supplemental Tables

Supplemental Table 1A. Percentage of reads mapped to different taxonomic levels in each rhinoceros species.

	All	Black Rhino	GOH Rhino	Sumatran Rhino	White Rhino
Phylum	0.9687	0.9762	0.9786	0.8781	0.9692
Class	0.8595	0.9138	0.8386	0.8683	0.8540
Order	0.7940	0.8668	0.7767	0.8467	0.7764
Family	0.6455	0.7407	0.6335	0.4787	0.6387
Genus	0.3096	0.3806	0.2635	0.2191	0.3227
Species	0.0064	0.0275	0.0020	0.0141	0.0017

Supplemental Table 1B. Percentage of ASVs mapped to taxonomic levels in each rhinoceros species.

	All	Black Rhino	GOH Rhino	Sumatran Rhino	White Rhino
Phylum	0.9609	0.9701	0.9614	0.9734	0.9583
Class	0.8736	0.8923	0.8785	0.9430	0.8717
Order	0.8011	0.8380	0.8156	0.8821	0.7978
Family	0.6353	0.6738	0.6517	0.7186	0.6336
Genus	0.3066	0.3348	0.3109	0.4030	0.3031
Species	0.0096	0.0192	0.0081	0.0304	0.0062

Supplemental Table 2. R^2 values for microbiome and metabolome samples.*

	R^2	ADONIS p-val	Dispersion p-val
Microbiome			
Species	0.49	0.001	0.001
Facility	0.17	0.001	0.001
Age	0.02	0.253	0.337
Sex	0.01	0.488	0.813
Metabolome			
Species	0.27	0.001	0.64
Facility	0.33	0.001	0.114
Age	0.01	0.634	0.563
Sex	0.02	0.332	0.731

*Values computed on the Euclidian distances after variance stabilizing transformation

Supplemental Table 3. Difference in ASV median location for IOD-susceptible versus IOD-resistant rhinoceros species.

ASV	Difference in location (95% CI)	p-value	BH-FDR p-value	Kingdom	Phylum	Class	Order	Family	Genus
ASV1007	11 (6; 14)	1.77E-05	1.77E-04	Bacteria	NA	NA	NA	NA	NA
ASV793	11.7 (8; 21)	1.81E-05	1.77E-04	Bacteria	Firmicutes	Clostridia	Clostridiales	Lachnospiraceae	NA
ASV1151	9.4 (5; 14)	1.81E-05	1.77E-04	Bacteria	Bacteroidetes	Bacteroidia	Bacteroidales	NA	NA
ASV190	78.5 (57; 92)	1.82E-05	1.77E-04	Bacteria	Firmicutes	Clostridia	Clostridiales	Ruminococcaceae	Ethanoligenens
ASV649	18.7 (11; 25)	1.82E-05	1.77E-04	Bacteria	Firmicutes	Clostridia	Clostridiales	Ruminococcaceae	Sporobacter
ASV712	14.5 (9; 21)	1.82E-05	1.77E-04	Bacteria	Firmicutes	Clostridia	Clostridiales	NA	NA
ASV611	19 (15; 30)	1.82E-05	1.77E-04	Bacteria	Firmicutes	Clostridia	Clostridiales	NA	NA
ASV1092	9 (6; 14)	1.82E-05	1.77E-04	Bacteria	Firmicutes	Clostridia	Clostridiales	Lachnospiraceae	Blautia
ASV351	30.5 (21; 51)	1.83E-05	1.77E-04	Bacteria	Firmicutes	Clostridia	Clostridiales	Ruminococcaceae	NA
ASV550	27.1 (20; 31)	1.83E-05	1.77E-04	Bacteria	Firmicutes	Clostridia	Clostridiales	NA	NA
ASV661	17.4 (12; 26)	1.83E-05	1.77E-04	Bacteria	Firmicutes	Clostridia	Clostridiales	Lachnospiraceae	NA
ASV283	42.8 (26; 81)	1.83E-05	1.77E-04	Bacteria	Firmicutes	Clostridia	Clostridiales	Ruminococcaceae	NA
ASV284	49.7 (22; 75)	1.83E-05	1.77E-04	Bacteria	Firmicutes	Clostridia	Clostridiales	Lachnospiraceae	NA
ASV481	22 (18; 39)	1.83E-05	1.77E-04	Bacteria	NA	NA	NA	NA	NA
ASV539	20.1 (9; 27)	1.83E-05	1.77E-04	Bacteria	Synergistetes	Synergistia	Synergistales	Synergistaceae	Synergistes
ASV175	43.4 (22; 71)	1.84E-05	1.77E-04	Bacteria	Bacteroidetes	NA	NA	NA	NA
ASV299	40 (33; 66)	1.84E-05	1.77E-04	Bacteria	Firmicutes	Clostridia	Clostridiales	Ruminococcaceae	NA
ASV304	34.6 (16; 86)	1.84E-05	1.77E-04	Bacteria	Firmicutes	Clostridia	Clostridiales	Lachnospiraceae	Clostridium_XlVa
ASV707	21.5 (12; 29)	1.84E-05	1.77E-04	Bacteria	Firmicutes	Clostridia	Clostridiales	Ruminococcaceae	NA
ASV76	139.8 (104; 220)	1.84E-05	1.77E-04	Bacteria	Spirochaetes	Spirochaetia	Spirochaetales	Spirochaetaceae	Treponema
ASV116	83.4 (35; 132)	1.84E-05	1.77E-04	Bacteria	Firmicutes	Clostridia	Clostridiales	Ruminococcaceae	Sporobacter
ASV134	92.6 (57; 112)	1.84E-05	1.77E-04	Bacteria	Firmicutes	Clostridia	Clostridiales	Lachnospiraceae	NA
ASV197	48 (40; 73)	1.84E-05	1.77E-04	Bacteria	Firmicutes	Clostridia	Clostridiales	Lachnospiraceae	NA
ASV246	62.5 (43; 82)	1.84E-05	1.77E-04	Bacteria	Firmicutes	Clostridia	Clostridiales	Ruminococcaceae	NA
ASV251	35 (25; 71)	1.84E-05	1.77E-04	Bacteria	Firmicutes	Clostridia	Clostridiales	Lachnospiraceae	NA
ASV300	51.1 (29; 67)	1.84E-05	1.77E-04	Bacteria	Firmicutes	Clostridia	Clostridiales	Lachnospiraceae	NA
ASV337	48.6 (23; 63)	1.84E-05	1.77E-04	Bacteria	Firmicutes	Negativicutes	Selenomonadales	Veillonellaceae	Selenomonas
ASV346	29.4 (20; 52)	1.84E-05	1.77E-04	Bacteria	Firmicutes	Clostridia	Clostridiales	NA	NA
ASV489	13.6 (8; 26)	1.84E-05	1.77E-04	Bacteria	Verrucomicrobia	Subdivision5	NA	NA	NA
ASV6	446 (339; 923)	1.85E-05	1.77E-04	Bacteria	Bacteroidetes	Bacteroidia	Bacteroidales	NA	NA
ASV9	363.8 (205; 657)	1.85E-05	1.77E-04	Bacteria	Bacteroidetes	NA	NA	NA	NA
ASV12	372.2 (323; 700)	1.85E-05	1.77E-04	Bacteria	Bacteroidetes	Bacteroidia	Bacteroidales	NA	NA
ASV17	376.8 (278; 542)	1.85E-05	1.77E-04	Bacteria	Firmicutes	Clostridia	Clostridiales	Lachnospiraceae	NA
ASV29	161.4 (121; 239)	1.85E-05	1.77E-04	Bacteria	Bacteroidetes	Bacteroidia	Bacteroidales	Prevotellaceae	Prevotella
ASV38	82.3 (53; 341)	1.85E-05	1.77E-04	Bacteria	Verrucomicrobia	Subdivision5	NA	NA	NA
ASV61	149.8 (60; 269)	1.85E-05	1.77E-04	Bacteria	Firmicutes	Negativicutes	Selenomonadales	Acidaminococcaceae	Acidaminococcus
ASV67	140.7 (87; 169)	1.85E-05	1.77E-04	Bacteria	Bacteroidetes	Bacteroidia	Bacteroidales	Prevotellaceae	Paraprevotella
ASV74	117.4 (50; 223)	1.85E-05	1.77E-04	Bacteria	Bacteroidetes	Bacteroidia	Bacteroidales	NA	NA
ASV82	149.9 (78; 199)	1.85E-05	1.77E-04	Bacteria	Firmicutes	Clostridia	Clostridiales	Lachnospiraceae	Clostridium_XlVa
ASV85	56.9 (37; 109)	1.85E-05	1.77E-04	Bacteria	Bacteroidetes	Bacteroidia	Bacteroidales	Prevotellaceae	Prevotella
ASV94	126.8 (31; 200)	1.85E-05	1.77E-04	Bacteria	Firmicutes	Clostridia	Clostridiales	Lachnospiraceae	NA
ASV142	92.9 (79; 127)	1.85E-05	1.77E-04	Bacteria	Firmicutes	Clostridia	Clostridiales	Ruminococcaceae	Sporobacter
ASV145	92.3 (64; 121)	1.85E-05	1.77E-04	Bacteria	Firmicutes	Clostridia	Clostridiales	Lachnospiraceae	NA
ASV223	59 (22; 88)	1.85E-05	1.77E-04	Bacteria	Bacteroidetes	Bacteroidia	Bacteroidales	Prevotellaceae	Alloprevotella
ASV256	54.1 (41; 66)	1.85E-05	1.77E-04	Bacteria	Firmicutes	Clostridia	Clostridiales	NA	NA
ASV258	61.8 (33; 80)	1.85E-05	1.77E-04	Bacteria	Firmicutes	Erysipelotrichia	Erysipelotrichales	Erysipelotrichaceae	NA
ASV240	53 (32; 89)	2.00E-05	1.81E-04	Bacteria	Verrucomicrobia	Subdivision5	NA	NA	NA
ASV44	157 (126; 205)	2.01E-05	1.81E-04	Bacteria	Bacteroidetes	Bacteroidia	Bacteroidales	NA	NA
ASV174	65 (53; 96)	2.01E-05	1.81E-04	Bacteria	Firmicutes	Clostridia	Clostridiales	Ruminococcaceae	NA
ASV482	17 (8; 32)	2.13E-05	1.82E-04	Bacteria	Proteobacteria	NA	NA	NA	NA
ASV390	33 (28; 43)	2.14E-05	1.82E-04	Bacteria	Firmicutes	Clostridia	Clostridiales	Ruminococcaceae	Sporobacter
ASV217	67 (46; 84)	2.14E-05	1.82E-04	Bacteria	Bacteroidetes	NA	NA	NA	NA

ASV	Difference in location (95% CI)	p-value	BH-FDR p-value	Kingdom	Phylum	Class	Order	Family	Genus
ASV354	35 (25; 50)	2.22E-05	1.85E-04	Bacteria	Firmicutes	NA	NA	NA	NA
ASV767	12 (10; 25)	2.41E-05	1.97E-04	Bacteria	Firmicutes	Clostridia	Clostridiales	Lachnospiraceae	NA
ASV313	49 (36; 62)	2.48E-05	1.97E-04	Bacteria	Firmicutes	Clostridia	Clostridiales	Ruminococcaceae	NA
ASV375	41 (23; 48)	2.63E-05	1.97E-04	Bacteria	Firmicutes	NA	NA	NA	NA
ASV176	75 (39; 120)	2.78E-05	1.97E-04	Bacteria	Firmicutes	NA	NA	NA	NA
ASV2053	3 (2; 6)	3.70E-05	1.97E-04	Bacteria	Firmicutes	NA	NA	NA	NA
ASV1055	9 (5; 14)	3.72E-05	1.97E-04	Bacteria	Firmicutes	Clostridia	Clostridiales	Ruminococcaceae	NA
ASV13	358 (192; 643)	3.78E-05	1.97E-04	Bacteria	Firmicutes	Clostridia	Clostridiales	Ruminococcaceae	Saccharofermentans
ASV170	66 (49; 92)	4.01E-05	1.97E-04	Bacteria	Firmicutes	Negativicutes	Selenomonadales	Veillonellaceae	Selenomonas
ASV205	67 (39; 91)	4.02E-05	1.97E-04	Bacteria	NA	NA	NA	NA	NA
ASV1118	6.5 (3; 14)	4.08E-05	1.97E-04	Bacteria	Firmicutes	Clostridia	Clostridiales	Lachnospiraceae	NA
ASV1058	8.4 (6; 16)	4.15E-05	1.97E-04	Bacteria	Actinobacteria	Actinobacteria	Coriobacteriales	Coriobacteriaceae	NA
ASV1020	10 (8; 14)	4.16E-05	1.97E-04	Bacteria	Firmicutes	Clostridia	Clostridiales	Ruminococcaceae	Sporobacter
ASV763	17.5 (13; 21)	4.17E-05	1.97E-04	Bacteria	Firmicutes	Clostridia	Clostridiales	Ruminococcaceae	NA
ASV1090	9 (7; 14)	4.20E-05	1.97E-04	Bacteria	Firmicutes	Clostridia	Clostridiales	Ruminococcaceae	Sporobacter
ASV606	20 (14; 28)	4.22E-05	1.97E-04	Bacteria	Firmicutes	Clostridia	Clostridiales	Ruminococcaceae	Sporobacter
ASV822	12.5 (7; 22)	4.22E-05	1.97E-04	Bacteria	Firmicutes	Clostridia	Clostridiales	Ruminococcaceae	Sporobacter
ASV708	9 (6; 23)	4.23E-05	1.97E-04	Bacteria	Planctomycetes	Planctomycetia	Planctomycetales	Planctomycetaceae	NA
ASV1037	8.9 (6; 13)	4.23E-05	1.97E-04	Bacteria	Firmicutes	Clostridia	Clostridiales	Ruminococcaceae	Oscillibacter
ASV285	48.3 (21; 67)	4.25E-05	1.97E-04	Bacteria	Firmicutes	Clostridia	Clostridiales	Ruminococcaceae	Acetivibrio
ASV572	14.6 (5; 35)	4.25E-05	1.97E-04	Bacteria	Armatimonadetes	Armatimonadetes_gp2	NA	NA	NA
ASV657	13 (7; 26)	4.26E-05	1.97E-04	Bacteria	Firmicutes	Clostridia	Clostridiales	Ruminococcaceae	Ruminococcus
ASV904	12.4 (9; 17)	4.26E-05	1.97E-04	Bacteria	Firmicutes	Clostridia	Clostridiales	Ruminococcaceae	Acetivibrio
ASV980	9 (7; 16)	4.26E-05	1.97E-04	Bacteria	Firmicutes	NA	NA	NA	NA
ASV266	59.5 (46; 77)	4.27E-05	1.97E-04	Bacteria	Firmicutes	Clostridia	Clostridiales	Syntrophomonadaceae	Pelospora
ASV418	28.4 (16; 34)	4.27E-05	1.97E-04	Bacteria	NA	NA	NA	NA	NA
ASV492	21.2 (12; 28)	4.27E-05	1.97E-04	Bacteria	Bacteroidetes	Bacteroidia	Bacteroidales	NA	NA
ASV598	21.6 (13; 32)	4.27E-05	1.97E-04	Bacteria	Firmicutes	Clostridia	Clostridiales	NA	NA
ASV716	16.4 (9; 30)	4.27E-05	1.97E-04	Bacteria	Firmicutes	Clostridia	Clostridiales	NA	NA
ASV727	14.1 (10; 22)	4.27E-05	1.97E-04	Bacteria	Firmicutes	Clostridia	Clostridiales	Lachnospiraceae	NA
ASV859	13.9 (8; 21)	4.27E-05	1.97E-04	Bacteria	Firmicutes	Clostridia	Clostridiales	Ruminococcaceae	Sporobacter
ASV124	59.5 (25; 104)	4.28E-05	1.97E-04	Bacteria	Chloroflexi	Anaerolineae	Anaerolineales	Anaerolineaceae	NA
ASV184	79 (42; 116)	4.28E-05	1.97E-04	Bacteria	Firmicutes	Clostridia	Clostridiales	Lachnospiraceae	Clostridium_XlVa
ASV280	45.3 (31; 69)	4.28E-05	1.97E-04	Bacteria	Bacteroidetes	Bacteroidia	Bacteroidales	Prevotellaceae	NA
ASV456	30 (20; 43)	4.28E-05	1.97E-04	Bacteria	Firmicutes	Clostridia	Clostridiales	Ruminococcaceae	Sporobacter
ASV583	15.9 (9; 20)	4.28E-05	1.97E-04	Bacteria	Firmicutes	Clostridia	Clostridiales	stridiales_Incertae_Sedis_	Mogibacterium
ASV706	13.9 (7; 24)	4.28E-05	1.97E-04	Bacteria	Verrucomicrobia	Subdivision5	NA	NA	NA
ASV870	13 (9; 18)	4.28E-05	1.97E-04	Bacteria	Firmicutes	Clostridia	Clostridiales	Lachnospiraceae	Acetitomaculum
ASV25	165 (97; 404)	4.29E-05	1.97E-04	Bacteria	Firmicutes	Clostridia	Clostridiales	Ruminococcaceae	NA
ASV40	214.1 (50; 298)	4.29E-05	1.97E-04	Bacteria	Firmicutes	Clostridia	Clostridiales	Lachnospiraceae	Clostridium_XlVa
ASV60	97.1 (29; 228)	4.29E-05	1.97E-04	Bacteria	Verrucomicrobia	Subdivision5	NA	NA	NA
ASV115	118.9 (65; 151)	4.29E-05	1.97E-04	Bacteria	Verrucomicrobia	Subdivision5	NA	NA	NA
ASV125	84.2 (25; 169)	4.30E-05	1.97E-04	Bacteria	Firmicutes	Clostridia	Clostridiales	Lachnospiraceae	Clostridium_XlVa
ASV488	29.2 (13; 42)	4.30E-05	1.97E-04	Bacteria	Firmicutes	Clostridia	Clostridiales	stridiales_Incertae_Sedis_	Anaerovorax
ASV425	26 (13; 40)	4.58E-05	2.08E-04	Bacteria	Firmicutes	NA	NA	NA	NA
ASV321	43 (33; 56)	6.00E-05	2.70E-04	Bacteria	Firmicutes	Clostridia	Clostridiales	Ruminococcaceae	Sporobacter
ASV1043	9 (6; 12)	6.10E-05	2.72E-04	Bacteria	Firmicutes	Clostridia	Clostridiales	Lachnospiraceae	NA
ASV120	80.5 (45; 145)	6.32E-05	2.79E-04	Bacteria	Firmicutes	Clostridia	Clostridiales	NA	NA
ASV853	7 (4; 19)	7.50E-05	3.06E-04	Bacteria	Firmicutes	Clostridia	Clostridiales	Lachnospiraceae	NA
ASV297	34 (23; 74)	7.69E-05	3.06E-04	Bacteria	Firmicutes	Clostridia	Clostridiales	Lachnospiraceae	NA
ASV233	48 (33; 77)	7.88E-05	3.06E-04	Bacteria	NA	NA	NA	NA	NA
ASV46	-537.2 (-752; -195)	8.29E-05	3.06E-04	Bacteria	Firmicutes	Clostridia	Clostridiales	Ruminococcaceae	Oscillibacter
ASV2060	3 (2; 5)	9.13E-05	3.06E-04	Bacteria	Firmicutes	Clostridia	Clostridiales	ostridiales_Incertae_Sedis_	Clostridium_XII
ASV1335	7.1 (5; 11)	9.26E-05	3.06E-04	Bacteria	Firmicutes	Clostridia	Clostridiales	Lachnospiraceae	NA

ASV	Difference in location (95% CI)	p-value	BH-FDR p-value	Kingdom	Phylum	Class	Order	Family	Genus
ASV320	47.2 (14; 72)	9.32E-05	3.06E-04	Bacteria	NA	NA	NA	NA	NA
ASV994	6.8 (3; 18)	9.34E-05	3.06E-04	Bacteria	NA	NA	NA	NA	NA
ASV1234	8.2 (4; 12)	9.34E-05	3.06E-04	Bacteria	NA	NA	NA	NA	NA
ASV739	13.8 (8; 23)	9.40E-05	3.06E-04	Bacteria	Firmicutes	Clostridia	Clostridiales	Ruminococcaceae	NA
ASV1331	7 (4; 11)	9.40E-05	3.06E-04	Bacteria	Firmicutes	Clostridia	Clostridiales	Lachnospiraceae	NA
ASV713	15.8 (6; 26)	9.42E-05	3.06E-04	Bacteria	Firmicutes	Clostridia	Clostridiales	stridiales_Incertae_Sedis_	Anaerovorax
ASV745	13.8 (6; 19)	9.42E-05	3.06E-04	Bacteria	Bacteroidetes	NA	NA	NA	NA
ASV806	13 (7; 21)	9.42E-05	3.06E-04	Bacteria	Firmicutes	Clostridia	Clostridiales	NA	NA
ASV1423	6.2 (4; 9)	9.42E-05	3.06E-04	Bacteria	Tenericutes	Mollicutes	Anaeroplasmatales	Anaeroplasmataceae	Anaeroplasma
ASV378	41.7 (6; 63)	9.44E-05	3.06E-04	Bacteria	Fibrobacteres	Fibrobacteria	Fibrobacterales	Fibrobacteraceae	Fibrobacter
ASV1202	9 (5; 13)	9.44E-05	3.06E-04	Bacteria	Firmicutes	Clostridia	Clostridiales	NA	NA
ASV827	13.5 (8; 20)	9.46E-05	3.06E-04	Bacteria	Firmicutes	Clostridia	Clostridiales	Ruminococcaceae	NA
ASV724	14.7 (12; 23)	9.48E-05	3.06E-04	Bacteria	Firmicutes	Clostridia	Clostridiales	NA	NA
ASV915	10.3 (5; 20)	9.48E-05	3.06E-04	Bacteria	Firmicutes	Erysipelotrichia	Erysipelotrichales	Erysipelotrichaceae	Holdemania
ASV1029	10.1 (3; 17)	9.48E-05	3.06E-04	Bacteria	Firmicutes	Clostridia	Clostridiales	Ruminococcaceae	NA
ASV263	34 (10; 75)	9.50E-05	3.06E-04	Bacteria	Firmicutes	Negativicutes	Selenomonadales	Veillonellaceae	Selenomonas
ASV267	24.7 (14; 34)	9.50E-05	3.06E-04	Bacteria	Proteobacteria	Deltaproteobacteria	Desulfovibrionales	NA	NA
ASV312	40.9 (28; 50)	9.50E-05	3.06E-04	Bacteria	Firmicutes	Clostridia	Clostridiales	Lachnospiraceae	NA
ASV335	38 (10; 52)	9.50E-05	3.06E-04	Bacteria	Firmicutes	Clostridia	Clostridiales	Ruminococcaceae	Saccharofermentans
ASV386	17.1 (10; 29)	9.50E-05	3.06E-04	Bacteria	Chloroflexi	Anaerolineae	Anaerolineales	Anaerolineaceae	NA
ASV413	20.1 (8; 43)	9.50E-05	3.06E-04	Bacteria	Chloroflexi	Anaerolineae	Anaerolineales	Anaerolineaceae	Ornatilinea
ASV431	36.5 (20; 43)	9.50E-05	3.06E-04	Bacteria	Firmicutes	Clostridia	Clostridiales	Ruminococcaceae	NA
ASV602	23.2 (16; 32)	9.50E-05	3.06E-04	Bacteria	Firmicutes	Clostridia	Clostridiales	stridiales_Incertae_Sedis_	Anaerovorax
ASV62	142.1 (76; 191)	9.52E-05	3.06E-04	Bacteria	Bacteroidetes	Bacteroidia	Bacteroidales	Porphyromonadaceae	NA
ASV103	95.7 (29; 172)	9.52E-05	3.06E-04	Bacteria	Firmicutes	Clostridia	Clostridiales	Ruminococcaceae	Clostridium_IV
ASV295	21.3 (10; 95)	9.52E-05	3.06E-04	Bacteria	Firmicutes	NA	NA	NA	NA
ASV429	19.8 (7; 58)	9.52E-05	3.06E-04	Bacteria	Firmicutes	Clostridia	Clostridiales	Lachnospiraceae	NA
ASV646	16.2 (8; 30)	9.52E-05	3.06E-04	Bacteria	Firmicutes	Clostridia	Clostridiales	Lachnospiraceae	NA
ASV70	131.8 (80; 211)	9.54E-05	3.06E-04	Bacteria	Firmicutes	Clostridia	Clostridiales	Ruminococcaceae	Sporobacter
ASV366	28.2 (11; 65)	9.54E-05	3.06E-04	Bacteria	Spirochaetes	Spirochaetia	Spirochaetales	Spirochaetaceae	Treponema
ASV433	28.3 (11; 55)	9.54E-05	3.06E-04	Bacteria	Verrucomicrobia	Subdivision5	NA	NA	NA
ASV476	27 (20; 36)	9.58E-05	3.06E-04	Bacteria	Firmicutes	Clostridia	Clostridiales	NA	NA
ASV33	154 (78; 322)	1.25E-04	3.98E-04	Bacteria	Firmicutes	Clostridia	Clostridiales	Ruminococcaceae	NA
ASV503	22 (13; 37)	1.30E-04	4.09E-04	Bacteria	Firmicutes	Clostridia	Clostridiales	stridiales_Incertae_Sedis_	Anaerovorax
ASV71	132 (73; 160)	1.33E-04	4.15E-04	Bacteria	Firmicutes	Clostridia	Clostridiales	Lachnospiraceae	Clostridium_XlVa
ASV21	271 (140; 389)	1.44E-04	4.49E-04	Bacteria	Firmicutes	Clostridia	Clostridiales	Ruminococcaceae	NA
ASV306	34 (12; 88)	1.52E-04	4.69E-04	Bacteria	Firmicutes	Clostridia	Clostridiales	Ruminococcaceae	NA
ASV130	88 (39; 123)	1.77E-04	5.10E-04	Bacteria	Firmicutes	Clostridia	Clostridiales	Lachnospiraceae	Acetatifactor
ASV298	50 (28; 68)	1.89E-04	5.10E-04	Bacteria	Firmicutes	Clostridia	Clostridiales	Lachnospiraceae	Clostridium_XlVa
ASV1578	3 (1; 6)	1.95E-04	5.10E-04	Bacteria	Firmicutes	Clostridia	Clostridiales	Ruminococcaceae	NA
ASV1377	6 (4; 9)	1.98E-04	5.10E-04	Bacteria	Firmicutes	Clostridia	Clostridiales	Lachnospiraceae	Acetatifactor
ASV1273	9.5 (7; 13)	1.99E-04	5.10E-04	Bacteria	Firmicutes	NA	NA	NA	NA
ASV846	11.8 (7; 22)	1.99E-04	5.10E-04	Bacteria	Bacteroidetes	NA	NA	NA	NA
ASV1004	7 (5; 16)	1.99E-04	5.10E-04	Bacteria	Bacteroidetes	NA	NA	NA	NA
ASV768	15 (8; 19)	2.00E-04	5.10E-04	Bacteria	Firmicutes	Clostridia	Clostridiales	Ruminococcaceae	NA
ASV803	16.1 (9; 24)	2.00E-04	5.10E-04	Bacteria	Firmicutes	Clostridia	Clostridiales	NA	NA
ASV1567	6 (3; 9)	2.00E-04	5.10E-04	Bacteria	Firmicutes	Clostridia	Clostridiales	NA	NA
ASV581	18 (4; 41)	2.00E-04	5.10E-04	Bacteria	Firmicutes	Clostridia	Clostridiales	Ruminococcaceae	NA
ASV625	10 (4; 28)	2.00E-04	5.10E-04	Bacteria	Firmicutes	Clostridia	Clostridiales	Eubacteriaceae	NA
ASV1279	7 (3; 10)	2.00E-04	5.10E-04	Bacteria	Firmicutes	Clostridia	Clostridiales	NA	NA
ASV439	13 (7; 33)	2.01E-04	5.10E-04	Bacteria	Bacteroidetes	Bacteroidia	Bacteroidales	Prevotellaceae	Prevotella
ASV988	10.5 (6; 18)	2.01E-04	5.10E-04	Bacteria	Bacteroidetes	Bacteroidia	Bacteroidales	Marinilabiliaceae	NA
ASV1103	8 (2; 15)	2.01E-04	5.10E-04	Bacteria	Firmicutes	Clostridia	Clostridiales	NA	NA
ASV1432	5.1 (2; 12)	2.01E-04	5.10E-04	Bacteria	Bacteroidetes	Bacteroidia	Bacteroidales	Rikenellaceae	Mucinivorans

ASV	Difference in location (95% CI)	p-value	BH-FDR p-value	Kingdom	Phylum	Class	Order	Family	Genus
ASV440	22.5 (17; 36)	2.01E-04	5.10E-04	Bacteria	Firmicutes	Clostridia	Clostridiales	NA	NA
ASV678	14.7 (8; 19)	2.01E-04	5.10E-04	Bacteria	Lentisphaerae	Oligosphaeria	Oligosphaerales	Oligosphaeraceae	Oligosphaera
ASV738	12 (10; 22)	2.01E-04	5.10E-04	Bacteria	Firmicutes	NA	NA	NA	NA
ASV839	11.4 (7; 19)	2.01E-04	5.10E-04	Bacteria	Firmicutes	Clostridia	Clostridiales	NA	NA
ASV1071	8.7 (2; 18)	2.01E-04	5.10E-04	Bacteria	Bacteroidetes	Bacteroidia	Bacteroidales	NA	NA
ASV1262	7.8 (4; 12)	2.01E-04	5.10E-04	Bacteria	Firmicutes	Clostridia	Clostridiales	NA	NA
ASV236	40.4 (19; 80)	2.02E-04	5.10E-04	Bacteria	NA	NA	NA	NA	NA
ASV419	23.9 (13; 42)	2.02E-04	5.10E-04	Bacteria	Verrucomicrobia	Subdivision5	NA	NA	NA
ASV509	19 (7; 41)	2.02E-04	5.10E-04	Bacteria	Firmicutes	NA	NA	NA	NA
ASV22	238.6 (99; 425)	2.02E-04	5.10E-04	Bacteria	Bacteroidetes	Bacteroidia	Bacteroidales	Porphyromonadaceae	NA
ASV108	123 (77; 151)	2.02E-04	5.10E-04	Bacteria	Firmicutes	Clostridia	Clostridiales	Ruminococcaceae	Sporobacter
ASV191	48 (14; 137)	2.02E-04	5.10E-04	Bacteria	Spirochaetes	Spirochaetia	Spirochaetales	Spirochaetaceae	Treponema
ASV19	130.2 (41; 720)	2.02E-04	5.10E-04	Bacteria	Bacteroidetes	Bacteroidia	Bacteroidales	Porphyromonadaceae	NA
ASV66	102 (33; 316)	2.02E-04	5.10E-04	Bacteria	Firmicutes	Clostridia	Clostridiales	Lachnospiraceae	NA
ASV86	100.1 (48; 197)	2.02E-04	5.10E-04	Bacteria	Bacteroidetes	NA	NA	NA	NA
ASV607	16 (7; 27)	2.09E-04	5.25E-04	Bacteria	Firmicutes	Clostridia	Clostridiales	NA	NA
ASV225	42 (25; 63)	2.11E-04	5.26E-04	Bacteria	Firmicutes	Clostridia	Clostridiales	stridiales_Incertae_Sedis_	Anaerovorax
ASV303	39 (17; 53)	2.31E-04	5.69E-04	Bacteria	Firmicutes	Clostridia	Clostridiales	Ruminococcaceae	NA
ASV645	19 (10; 25)	2.31E-04	5.69E-04	Bacteria	Firmicutes	Clostridia	Clostridiales	stridiales_Incertae_Sedis_	Anaerovorax
ASV112	-152 (-452; -69)	2.36E-04	5.77E-04	Bacteria	Firmicutes	Clostridia	Clostridiales	NA	NA
ASV1230	7 (4; 12)	3.00E-04	7.31E-04	Bacteria	Proteobacteria	Gammaproteobacteria	NA	NA	NA
ASV42	245 (108; 275)	3.04E-04	7.37E-04	Bacteria	Verrucomicrobia	Subdivision5	NA	NA	NA
ASV553	-36 (-127; -20)	3.36E-04	8.09E-04	Bacteria	Firmicutes	Clostridia	Clostridiales	Lachnospiraceae	NA
ASV59	104.5 (58; 213)	3.50E-04	8.10E-04	Bacteria	Firmicutes	Clostridia	Clostridiales	Lachnospiraceae	NA
ASV362	31.2 (15; 60)	3.67E-04	8.10E-04	Bacteria	Firmicutes	Clostridia	Clostridiales	Ruminococcaceae	NA
ASV1871	5 (2; 6)	3.90E-04	8.10E-04	Bacteria	Firmicutes	Clostridia	Clostridiales	NA	NA
ASV847	7 (1; 14)	3.93E-04	8.10E-04	Bacteria	Bacteroidetes	NA	NA	NA	NA
ASV1873	3 (2; 5)	3.95E-04	8.10E-04	Bacteria	NA	NA	NA	NA	NA
ASV1703	5 (3; 7)	4.02E-04	8.10E-04	Bacteria	Firmicutes	Clostridia	Clostridiales	Lachnospiraceae	NA
ASV1077	9.8 (5; 14)	4.03E-04	8.10E-04	Bacteria	Firmicutes	Clostridia	Clostridiales	NA	NA
ASV1536	4 (2; 6)	4.04E-04	8.10E-04	Bacteria	Firmicutes	Clostridia	Clostridiales	Ruminococcaceae	NA
ASV1555	5 (3; 9)	4.05E-04	8.10E-04	Bacteria	Firmicutes	Clostridia	Clostridiales	NA	NA
ASV1094	6 (4; 15)	4.06E-04	8.10E-04	Bacteria	Firmicutes	Clostridia	Clostridiales	Ruminococcaceae	NA
ASV144	70 (46; 122)	4.06E-04	8.10E-04	Bacteria	Firmicutes	Clostridia	Clostridiales	NA	NA
ASV1190	5.8 (3; 13)	4.06E-04	8.10E-04	Bacteria	Actinobacteria	Actinobacteria	Coriobacteriales	Coriobacteriaceae	Parvibacter
ASV908	10.7 (4; 19)	4.07E-04	8.10E-04	Bacteria	Firmicutes	Clostridia	Clostridiales	Lachnospiraceae	NA
ASV981	6 (2; 19)	4.07E-04	8.10E-04	Bacteria	Proteobacteria	Deltaproteobacteria	NA	NA	NA
ASV1124	8.3 (4; 15)	4.07E-04	8.10E-04	Bacteria	Firmicutes	Clostridia	Clostridiales	Lachnospiraceae	NA
ASV1634	3.4 (1; 7)	4.07E-04	8.10E-04	Bacteria	Firmicutes	Clostridia	Clostridiales	NA	NA
ASV1520	5 (1; 11)	4.08E-04	8.10E-04	Bacteria	Firmicutes	Clostridia	Clostridiales	NA	NA
ASV703	15.9 (8; 24)	4.08E-04	8.10E-04	Bacteria	Firmicutes	Clostridia	Clostridiales	NA	NA
ASV746	13 (3; 21)	4.08E-04	8.10E-04	Bacteria	Verrucomicrobia	Subdivision5	NA	NA	NA
ASV635	6 (2; 28)	4.09E-04	8.10E-04	Bacteria	Firmicutes	Clostridia	Clostridiales	NA	NA
ASV925	12.5 (5; 20)	4.09E-04	8.10E-04	Bacteria	Bacteroidetes	Bacteroidia	Bacteroidales	Rikenellaceae	Mucinivorans
ASV1061	6.6 (3; 16)	4.09E-04	8.10E-04	Bacteria	Firmicutes	Clostridia	Clostridiales	NA	NA
ASV1248	6.5 (3; 11)	4.09E-04	8.10E-04	Bacteria	Chloroflexi	Anaerolineae	Anaerolineales	Anaerolineaceae	Ornatilinea
ASV104	27 (3; 106)	4.10E-04	8.10E-04	Bacteria	Firmicutes	Clostridia	Clostridiales	Ruminococcaceae	Faecalibacterium
ASV324	20.4 (8; 80)	4.10E-04	8.10E-04	Bacteria	Actinobacteria	Actinobacteria	Coriobacteriales	Coriobacteriaceae	NA
ASV381	16.6 (5; 83)	4.10E-04	8.10E-04	Bacteria	Bacteroidetes	NA	NA	NA	NA
ASV733	9 (3; 38)	4.10E-04	8.10E-04	Bacteria	Firmicutes	Clostridia	Clostridiales	NA	NA
ASV868	9.1 (5; 17)	4.10E-04	8.10E-04	Bacteria	Firmicutes	Clostridia	Clostridiales	Lachnospiraceae	NA
ASV886	8.3 (4; 15)	4.10E-04	8.10E-04	Bacteria	Bacteroidetes	Bacteroidia	Bacteroidales	Prevotellaceae	Paraprevotella
ASV250	31.1 (10; 85)	4.11E-04	8.10E-04	Bacteria	Firmicutes	Clostridia	Clostridiales	Lachnospiraceae	NA
ASV328	38.5 (24; 58)	4.11E-04	8.10E-04	Bacteria	NA	NA	NA	NA	NA

ASV	Difference in location (95% CI)	p-value	BH-FDR p-value	Kingdom	Phylum	Class	Order	Family	Genus
ASV359	29.2 (11; 62)	4.11E-04	8.10E-04	Bacteria	Firmicutes	Clostridia	Clostridiales	Ruminococcaceae	NA
ASV392	35.1 (23; 47)	4.11E-04	8.10E-04	Bacteria	Firmicutes	Clostridia	Clostridiales	Ruminococcaceae	Acetivibrio
ASV432	26.2 (10; 60)	4.11E-04	8.10E-04	Bacteria	Firmicutes	Clostridia	Clostridiales	NA	NA
ASV567	19 (7; 30)	4.11E-04	8.10E-04	Bacteria	Firmicutes	Clostridia	Clostridiales	Ruminococcaceae	NA
ASV805	15.3 (7; 24)	4.11E-04	8.10E-04	Bacteria	Firmicutes	Clostridia	Clostridiales	Lachnospiraceae	Clostridium_XlVa
ASV1	1432 (285; 2562)	4.11E-04	8.10E-04	Bacteria	Firmicutes	Clostridia	Clostridiales	Ruminococcaceae	Ruminococcus
ASV91	118.6 (49; 234)	4.11E-04	8.10E-04	Bacteria	Firmicutes	Clostridia	Clostridiales	NA	NA
ASV123	98.1 (46; 155)	4.11E-04	8.10E-04	Bacteria	Bacteroidetes	NA	NA	NA	NA
ASV154	83.9 (20; 137)	4.11E-04	8.10E-04	Bacteria	Firmicutes	NA	NA	NA	NA
ASV160	87.1 (42; 151)	4.11E-04	8.10E-04	Bacteria	Verrucomicrobia	Subdivision5	NA	NA	NA
ASV126	66 (18; 108)	4.23E-04	8.28E-04	Bacteria	Firmicutes	Clostridia	Clostridiales	Ruminococcaceae	Sporobacter
ASV199	36 (7; 101)	4.43E-04	8.64E-04	Bacteria	Spirochaetes	Spirochaetia	Spirochaetales	Spirochaetaceae	Treponema
ASV468	21 (11; 36)	4.60E-04	8.94E-04	Bacteria	Bacteroidetes	Bacteroidia	Bacteroidales	NA	NA
ASV277	58 (23; 71)	4.62E-04	8.94E-04	Bacteria	Firmicutes	Erysipelotrichia	Erysipelotrichales	Erysipelotrichaceae	NA
ASV379	26 (18; 40)	4.74E-04	9.13E-04	Bacteria	Firmicutes	Clostridia	Clostridiales	Lachnospiraceae	NA
ASV53	152.5 (94; 207)	5.14E-04	9.85E-04	Bacteria	Firmicutes	Clostridia	Clostridiales	Ruminococcaceae	NA
ASV206	51 (39; 81)	5.52E-04	0.001054342	Bacteria	Firmicutes	Clostridia	Clostridiales	Ruminococcaceae	Oscillibacter
ASV917	13 (6; 18)	5.77E-04	0.001094928	Bacteria	Bacteroidetes	Bacteroidia	Bacteroidales	NA	NA
ASV2	1281 (541; 1746)	5.78E-04	0.001094928	Bacteria	Firmicutes	NA	NA	NA	NA
ASV1359	7 (3; 9)	5.82E-04	0.001097354	Bacteria	Firmicutes	Clostridia	Clostridiales	Ruminococcaceae	NA
ASV99	55 (25; 222)	6.22E-04	0.00116688	Bacteria	Firmicutes	Clostridia	Clostridiales	Lachnospiraceae	NA
ASV758	13 (8; 20)	6.53E-04	0.001180206	Bacteria	Firmicutes	Clostridia	Clostridiales	Lachnospiraceae	NA
ASV81	49.2 (26; 161)	6.59E-04	0.001180206	Bacteria	Bacteroidetes	Bacteroidia	Bacteroidales	Prevotellaceae	Alloprevotella
ASV942	12 (4; 13)	7.33E-04	0.001180206	Bacteria	Firmicutes	Clostridia	Clostridiales	Ruminococcaceae	Sporobacter
ASV63	102 (66; 208)	7.49E-04	0.001180206	Bacteria	Firmicutes	NA	NA	NA	NA
ASV500	17 (8; 24)	7.73E-04	0.001180206	Bacteria	Firmicutes	Clostridia	Clostridiales	Lachnospiraceae	NA
ASV717	14 (11; 20)	7.73E-04	0.001180206	Bacteria	Firmicutes	Clostridia	Clostridiales	Ruminococcaceae	NA
ASV2090	2.7 (1; 4)	7.81E-04	0.001180206	Bacteria	Firmicutes	Erysipelotrichia	Erysipelotrichales	Erysipelotrichaceae	NA
ASV161	48 (23; 80)	7.83E-04	0.001180206	Bacteria	Bacteroidetes	Bacteroidia	Bacteroidales	Porphyromonadaceae	NA
ASV1950	2 (1; 6)	7.84E-04	0.001180206	Bacteria	Firmicutes	Clostridia	Clostridiales	Clostridiaceae_1	NA
ASV2015	3 (1; 6)	7.84E-04	0.001180206	Bacteria	Actinobacteria	Actinobacteria	Coriobacteriales	Coriobacteriaceae	NA
ASV2009	3.3 (2; 5)	7.88E-04	0.001180206	Bacteria	Firmicutes	Erysipelotrichia	Erysipelotrichales	Erysipelotrichaceae	NA
ASV669	15.1 (2; 28)	7.89E-04	0.001180206	Bacteria	Firmicutes	Clostridia	Clostridiales	Lachnospiraceae	NA
ASV1245	9 (2; 11)	7.89E-04	0.001180206	Bacteria	Actinobacteria	Actinobacteria	Coriobacteriales	Coriobacteriaceae	Parvibacter
ASV2387	3 (1; 5)	7.89E-04	0.001180206	Bacteria	Firmicutes	Clostridia	Clostridiales	Ruminococcaceae	Papillibacter
ASV1421	3 (1; 8)	7.91E-04	0.001180206	Bacteria	Firmicutes	Clostridia	Clostridiales	Ruminococcaceae	Clostridium_IV
ASV950	3 (1; 19)	7.92E-04	0.001180206	Bacteria	Bacteroidetes	Bacteroidia	Bacteroidales	NA	NA
ASV1379	8.1 (4; 12)	7.92E-04	0.001180206	Bacteria	Firmicutes	Clostridia	Clostridiales	stridiales_Incertae_Sedis_	Anaerovorax
ASV1057	8 (6; 14)	7.93E-04	0.001180206	Bacteria	Planctomycetes	Planctomycetia	Planctomycetales	Planctomycetaceae	Pirellula
ASV1326	6 (3; 9)	7.93E-04	0.001180206	Bacteria	Firmicutes	Clostridia	Clostridiales	Ruminococcaceae	NA
ASV2087	2.2 (1; 8)	7.93E-04	0.001180206	Bacteria	Firmicutes	Clostridia	Clostridiales	Lachnospiraceae	Anaerosporobacter
ASV564	13.6 (4; 20)	7.95E-04	0.001180206	Bacteria	Firmicutes	Clostridia	Clostridiales	Lachnospiraceae	NA
ASV923	12.5 (9; 18)	7.95E-04	0.001180206	Bacteria	Firmicutes	Clostridia	Clostridiales	Ruminococcaceae	NA
ASV1226	7 (4; 13)	7.95E-04	0.001180206	Bacteria	Verrucomicrobia	Subdivision5	NA	NA	NA
ASV1281	6.1 (1; 11)	7.95E-04	0.001180206	Bacteria	Firmicutes	Erysipelotrichia	Erysipelotrichales	Erysipelotrichaceae	Bulleidia
ASV1013	5.5 (1; 10)	7.96E-04	0.001180206	Bacteria	Bacteroidetes	Bacteroidia	Bacteroidales	Prevotellaceae	Prevotella
ASV618	7 (3; 16)	7.99E-04	0.001180206	Bacteria	Bacteroidetes	Bacteroidia	Bacteroidales	Prevotellaceae	Prevotella
ASV910	11.1 (4; 20)	7.99E-04	0.001180206	Bacteria	Firmicutes	Clostridia	Clostridiales	NA	NA
ASV943	12.8 (1; 29)	7.99E-04	0.001180206	Bacteria	Firmicutes	Clostridia	Clostridiales	Ruminococcaceae	Oscillibacter
ASV1249	5.7 (1; 8)	7.99E-04	0.001180206	Bacteria	Firmicutes	Clostridia	Clostridiales	Lachnospiraceae	NA
ASV1495	6 (3; 11)	7.99E-04	0.001180206	Bacteria	Firmicutes	Clostridia	Clostridiales	Ruminococcaceae	NA
ASV1666	5 (2; 8)	7.99E-04	0.001180206	Bacteria	Firmicutes	Clostridia	Clostridiales	Ruminococcaceae	NA
ASV376	36.1 (5; 61)	8.00E-04	0.001180206	Bacteria	Bacteroidetes	Bacteroidia	Bacteroidales	NA	NA
ASV515	6 (1; 26)	8.00E-04	0.001180206	Bacteria	Bacteroidetes	Bacteroidia	Bacteroidales	Porphyromonadaceae	NA

ASV	Difference in location (95% CI)	p-value	BH-FDR p-value	Kingdom	Phylum	Class	Order	Family	Genus
ASV576	16.5 (6; 25)	8.00E-04	0.001180206	Bacteria	Firmicutes	Clostridia	Clostridiales	Lachnospiraceae	NA
ASV673	15.5 (13; 24)	8.00E-04	0.001180206	Bacteria	Firmicutes	Clostridia	Clostridiales	Ruminococcaceae	NA
ASV736	18.6 (5; 30)	8.00E-04	0.001180206	Bacteria	Firmicutes	Negativicutes	Selenomonadales	Veillonellaceae	NA
ASV1102	8.4 (4; 13)	8.00E-04	0.001180206	Bacteria	Actinobacteria	Actinobacteria	Coriobacteriales	Coriobacteriaceae	Parvibacter
ASV101	49.1 (18; 174)	8.02E-04	0.001180206	Bacteria	Firmicutes	Clostridia	Clostridiales	Lachnospiraceae	NA
ASV109	81.4 (27; 172)	8.02E-04	0.001180206	Bacteria	Firmicutes	Clostridia	Clostridiales	Ruminococcaceae	Clostridium_IV
ASV383	7.7 (2; 55)	8.02E-04	0.001180206	Bacteria	Verrucomicrobia	Subdivision5	NA	NA	NA
ASV398	17.5 (4; 55)	8.02E-04	0.001180206	Bacteria	Bacteroidetes	Bacteroidia	Bacteroidales	Prevotellaceae	Prevotella
ASV404	42.5 (9; 49)	8.02E-04	0.001180206	Bacteria	Bacteroidetes	NA	NA	NA	NA
ASV423	33.8 (14; 52)	8.02E-04	0.001180206	Bacteria	Firmicutes	Clostridia	Clostridiales	Ruminococcaceae	NA
ASV449	18.9 (9; 36)	8.02E-04	0.001180206	Bacteria	Firmicutes	Clostridia	Clostridiales	Ruminococcaceae	NA
ASV467	14 (2; 49)	8.02E-04	0.001180206	Bacteria	Firmicutes	Clostridia	Clostridiales	Ruminococcaceae	NA
ASV480	19 (5; 32)	8.02E-04	0.001180206	Bacteria	Verrucomicrobia	Subdivision5	NA	NA	NA
ASV522	23.9 (5; 42)	8.02E-04	0.001180206	Bacteria	Verrucomicrobia	Subdivision5	NA	NA	NA
ASV545	9.1 (4; 28)	8.02E-04	0.001180206	Bacteria	Firmicutes	Clostridia	Clostridiales	Ruminococcaceae	NA
ASV636	11 (4; 35)	8.02E-04	0.001180206	Bacteria	Firmicutes	Clostridia	Clostridiales	Ruminococcaceae	Sporobacter
ASV654	20.7 (7; 37)	8.02E-04	0.001180206	Bacteria	Firmicutes	Clostridia	Clostridiales	Lachnospiraceae	NA
ASV959	6.7 (2; 18)	8.02E-04	0.001180206	Bacteria	Firmicutes	Clostridia	Clostridiales	stridiales_Incertae_Sedis_	Anaerovorax
ASV968	11.7 (4; 19)	8.02E-04	0.001180206	Bacteria	Firmicutes	Clostridia	Clostridiales	Ruminococcaceae	NA
ASV16	91.3 (27; 139)	8.03E-04	0.001180206	Bacteria	Bacteroidetes	NA	NA	NA	NA
ASV32	37 (12; 542)	8.03E-04	0.001180206	Bacteria	Firmicutes	Clostridia	Clostridiales	Ruminococcaceae	NA
ASV35	45.5 (1; 372)	8.03E-04	0.001180206	Bacteria	Fibrobacteres	Fibrobacteria	Fibrobacterales	Fibrobacteraceae	Fibrobacter
ASV55	198.8 (5; 327)	8.03E-04	0.001180206	Bacteria	Firmicutes	Clostridia	Clostridiales	Lachnospiraceae	NA
ASV84	43.8 (12; 120)	8.03E-04	0.001180206	Bacteria	Verrucomicrobia	Subdivision5	NA	NA	NA
ASV177	24.3 (6; 146)	8.03E-04	0.001180206	Bacteria	Firmicutes	Clostridia	Clostridiales	Ruminococcaceae	Intestinimonas
ASV216	24.4 (6; 110)	8.03E-04	0.001180206	Bacteria	Bacteroidetes	NA	NA	NA	NA
ASV243	35 (2; 107)	8.03E-04	0.001180206	Bacteria	Spirochaetes	Spirochaetia	Spirochaetales	Spirochaetaceae	Treponema
ASV407	14 (2; 74)	8.03E-04	0.001180206	Bacteria	Verrucomicrobia	Subdivision5	NA	NA	NA
ASV494	10.9 (4; 52)	8.03E-04	0.001180206	Bacteria	Firmicutes	Clostridia	Clostridiales	Lachnospiraceae	NA
ASV562	24.1 (13; 36)	8.03E-04	0.001180206	Bacteria	Firmicutes	Clostridia	Clostridiales	NA	NA
ASV574	18.2 (8; 34)	8.03E-04	0.001180206	Bacteria	Firmicutes	Clostridia	Clostridiales	NA	NA
ASV801	10.7 (4; 21)	8.03E-04	0.001180206	Bacteria	Firmicutes	Clostridia	Clostridiales	Ruminococcaceae	Clostridium_IV
ASV45	107.7 (42; 272)	8.18E-04	0.001198765	Bacteria	Spirochaetes	Spirochaetia	Spirochaetales	Spirochaetaceae	Treponema
ASV680	9 (3; 18)	8.82E-04	0.001288429	Bacteria	Firmicutes	Clostridia	Clostridiales	Lachnospiraceae	Acetatifactor
ASV1552	5 (1; 7)	8.85E-04	0.001288429	Bacteria	Firmicutes	NA	NA	NA	NA
ASV368	32 (13; 50)	9.34E-04	0.001355204	Bacteria	Firmicutes	Clostridia	Clostridiales	NA	NA
ASV396	28 (17; 47)	9.78E-04	0.001413954	Bacteria	Firmicutes	Clostridia	Clostridiales	NA	NA
ASV34	178 (36; 343)	0.00106681	0.001537461	Bacteria	Firmicutes	Clostridia	Clostridiales	Lachnospiraceae	NA
ASV742	10 (5; 19)	0.001078367	0.001549054	Bacteria	Firmicutes	Clostridia	Clostridiales	NA	NA
ASV1123	7 (3; 12)	0.001149254	0.001645523	Bacteria	Bacteroidetes	Bacteroidia	Bacteroidales	NA	NA
ASV1160	7 (3; 11)	0.001186564	0.001693445	Bacteria	Firmicutes	Clostridia	Clostridiales	Lachnospiraceae	NA
ASV278	39 (15; 75)	0.001193379	0.001697677	Bacteria	Firmicutes	Clostridia	Clostridiales	Ruminococcaceae	NA
ASV229	50 (15; 79)	0.001237583	0.001754901	Bacteria	Firmicutes	Clostridia	Clostridiales	Lachnospiraceae	NA
ASV1397	6 (1; 11)	0.00124533	0.001760226	Bacteria	Firmicutes	Clostridia	Clostridiales	NA	NA
ASV339	42 (22; 49)	0.001337721	0.001884012	Bacteria	Firmicutes	Clostridia	Clostridiales	stridiales_Incertae_Sedis_	Anaerovorax
ASV566	13 (6; 35)	0.001341451	0.001884012	Bacteria	Verrucomicrobia	Subdivision5	NA	NA	NA
ASV51	74 (13; 172)	0.001459467	0.002043254	Bacteria	Bacteroidetes	Bacteroidia	Bacteroidales	Porphyromonadaceae	NA
ASV173	43 (8; 109)	0.001589269	0.002217935	Bacteria	Bacteroidetes	NA	NA	NA	NA
ASV927	7 (4; 9)	0.001701317	0.002366817	Bacteria	Firmicutes	Clostridia	Clostridiales	Lachnospiraceae	NA
ASV1999	3 (1; 5)	0.001711164	0.00237303	Bacteria	Actinobacteria	Actinobacteria	Coriobacteriales	Coriobacteriaceae	NA
ASV590	15 (3; 33)	0.002012566	0.002782262	Bacteria	Firmicutes	Clostridia	Clostridiales	Lachnospiraceae	NA
ASV700	-10.1 (-82; -5)	0.00203393	0.00280301	Bacteria	Firmicutes	Clostridia	NA	NA	NA
ASV372	26.2 (11; 41)	0.002286687	0.003141523	Bacteria	Firmicutes	Clostridia	Clostridiales	Ruminococcaceae	NA
ASV309	-77.1 (-117; -43)	0.002335057	0.003198013	Bacteria	Firmicutes	Clostridia	Clostridiales	NA	NA

ASV	Difference in location (95% CI)	p-value	BH-FDR p-value	Kingdom	Phylum	Class	Order	Family	Genus
ASV402	24 (6; 39)	0.00256952	0.003508231	Bacteria	Firmicutes	NA	NA	NA	NA
ASV149	56 (20; 82)	0.00295823	0.004026479	Bacteria	Firmicutes	Clostridia	Clostridiales	NA	NA
ASV31	176.8 (67; 337)	0.003005444	0.004078156	Bacteria	Firmicutes	Clostridia	Clostridiales	Lachnospiraceae	NA
ASV2172	2 (0; 5)	0.003176435	0.004296956	Bacteria	Firmicutes	Erysipelotrichia	Erysipelotrichales	Erysipelotrichaceae	NA
ASV918	-32 (-44; -10)	0.003404826	0.004591829	Bacteria	Firmicutes	Clostridia	Clostridiales	Ruminococcaceae	Sporobacter
ASV1211	6.9 (1; 8)	0.004005574	0.005385543	Bacteria	Firmicutes	Clostridia	Clostridiales	Lachnospiraceae	NA
ASV97	74 (26; 153)	0.004075851	0.005463375	Bacteria	Spirochaetes	Spirochaetia	Spirochaetales	Spirochaetaceae	Treponema
ASV164	45 (31; 77)	0.004239671	0.005665742	Bacteria	Bacteroidetes	Flavobacteriia	Flavobacteriales	Flavobacteriaceae	NA
ASV2088	3 (0; 5)	0.004389623	0.00584841	Bacteria	Firmicutes	Clostridia	Clostridiales	Ruminococcaceae	ydrogenoanaerobacteriun
ASV536	13 (2; 40)	0.004842464	0.006432309	Bacteria	Firmicutes	Erysipelotrichia	Erysipelotrichales	Erysipelotrichaceae	NA
ASV1813	3 (1; 4)	0.005234516	0.006926961	Bacteria	Bacteroidetes	Bacteroidia	Bacteroidales	Bacteroidaceae	Anaerorhabdus
ASV196	31.8 (0; 101)	0.00524627	0.006926961	Bacteria	Verrucomicrobia	Subdivision5	NA	NA	NA
ASV210	36 (7; 61)	0.005311575	0.006992252	Bacteria	Firmicutes	Clostridia	Clostridiales	Ruminococcaceae	NA
ASV65	97 (29; 176)	0.005351747	0.007024168	Bacteria	Spirochaetes	Spirochaetia	Spirochaetales	Spirochaetaceae	Treponema
ASV947	5 (0; 14)	0.007997751	0.010465899	Bacteria	Firmicutes	Clostridia	Clostridiales	Lachnospiraceae	NA
ASV2097	2 (0; 5)	0.008046582	0.010498647	Bacteria	Proteobacteria	NA	NA	NA	NA
ASV952	8 (0; 17)	0.00818451	0.010647106	Bacteria	Firmicutes	Clostridia	Clostridiales	NA	NA
ASV890	5 (2; 14)	0.008344003	0.010822662	Bacteria	Firmicutes	Clostridia	Clostridiales	Ruminococcaceae	NA
ASV1297	4 (0; 13)	0.00861048	0.011135548	Bacteria	Firmicutes	Clostridia	Clostridiales	Ruminococcaceae	NA
ASV518	6 (0; 20)	0.008864975	0.011431153	Bacteria	Firmicutes	NA	NA	NA	NA
ASV427	17 (0; 36)	0.008892042	0.011432626	Bacteria	Firmicutes	Clostridia	Clostridiales	Lachnospiraceae	NA
ASV389	21 (10; 50)	0.009098881	0.011664554	Bacteria	Bacteroidetes	NA	NA	NA	NA
ASV252	21.9 (0; 91)	0.010933464	0.013975819	Bacteria	Spirochaetes	Spirochaetia	Spirochaetales	Spirochaetaceae	Treponema
ASV192	58 (1; 81)	0.012325483	0.015709647	Bacteria	Firmicutes	Clostridia	Clostridiales	Ruminococcaceae	NA
ASV879	8.5 (0; 23)	0.012575141	0.015981663	Bacteria	Firmicutes	Clostridia	Clostridiales	Ruminococcaceae	NA
ASV1325	6 (3; 8)	0.012970717	0.01643703	Bacteria	Firmicutes	Clostridia	Clostridiales	NA	NA
ASV511	3 (0; 12)	0.014432228	0.018218026	Bacteria	Firmicutes	Clostridia	Clostridiales	NA	NA
ASV24	53 (0; 340)	0.014458751	0.018218026	Bacteria	Firmicutes	Clostridia	Clostridiales	Ruminococcaceae	NA
ASV655	11.8 (2; 27)	0.0146418	0.018396107	Bacteria	Firmicutes	Clostridia	Clostridiales	Lachnospiraceae	Lachnobacterium
ASV858	11 (3; 17)	0.015522467	0.019447182	Bacteria	Firmicutes	Clostridia	Clostridiales	stridiales_Incertae_Sedis_	NA
ASV238	30.3 (0; 85)	0.016095718	0.020108249	Bacteria	Firmicutes	Clostridia	Clostridiales	Ruminococcaceae	Sporobacter
ASV1420	2 (0; 10)	0.016292611	0.020296728	Bacteria	Firmicutes	Clostridia	Clostridiales	Ruminococcaceae	Intestinimonas
ASV454	13.6 (4; 30)	0.016738258	0.02079316	Bacteria	Firmicutes	Clostridia	Clostridiales	Lachnospiraceae	NA
ASV105	84 (45; 138)	0.016806176	0.020818887	Bacteria	Firmicutes	Clostridia	Clostridiales	Ruminococcaceae	NA
ASV5	424 (114; 1033)	0.01701062	0.021013119	Bacteria	Firmicutes	Clostridia	Clostridiales	NA	NA
ASV552	13 (3; 17)	0.017748305	0.021863135	Bacteria	Firmicutes	Clostridia	Clostridiales	Ruminococcaceae	Sporobacter
ASV52	84 (20; 127)	0.018787863	0.023079241	Bacteria	Firmicutes	Clostridia	Clostridiales	Ruminococcaceae	Anaerotruncus
ASV441	19 (2; 30)	0.020198667	0.024743367	Bacteria	Firmicutes	Clostridia	Clostridiales	Ruminococcaceae	Sporobacter
ASV855	9 (3; 13)	0.02240902	0.027375007	Bacteria	Firmicutes	Erysipelotrichia	Erysipelotrichales	Erysipelotrichaceae	NA
ASV1018	8 (0; 14)	0.022738084	0.027700263	Bacteria	Firmicutes	Clostridia	Clostridiales	Ruminococcaceae	NA
ASV956	7 (0; 16)	0.022813559	0.027715646	Bacteria	Bacteroidetes	Bacteroidia	Bacteroidales	NA	NA
ASV595	15 (0; 26)	0.022938439	0.027790801	Bacteria	Firmicutes	Clostridia	Clostridiales	NA	NA
ASV96	72.1 (9; 117)	0.023565733	0.02847257	Bacteria	Firmicutes	Clostridia	Clostridiales	NA	NA
ASV18	36.7 (2; 185)	0.024076722	0.029010477	Bacteria	Firmicutes	Clostridia	Clostridiales	Ruminococcaceae	Ruminococcus
ASV648	5 (0; 34)	0.024332512	0.029238795	Bacteria	Verrucomicrobia	Subdivision5	NA	NA	NA
ASV1796	3 (0; 8)	0.024438649	0.029286534	Bacteria	Firmicutes	Clostridia	Clostridiales	NA	NA
ASV1426	4 (0; 8)	0.026469352	0.031634103	Bacteria	Firmicutes	Clostridia	NA	NA	NA
ASV293	22 (8; 36)	0.027659084	0.032966637	Bacteria	Firmicutes	Clostridia	Clostridiales	Ruminococcaceae	Acetivibrio
ASV466	-43 (-66; 0)	0.027883568	0.033144619	Bacteria	Firmicutes	Clostridia	Clostridiales	Ruminococcaceae	NA
ASV326	-31 (-68; 0)	0.031224279	0.037015879	Bacteria	Firmicutes	Clostridia	Clostridiales	Lachnospiraceae	Pseudobutyrivibrio
ASV310	16 (1; 38)	0.034632227	0.040945877	Bacteria	Firmicutes	Clostridia	Clostridiales	Lachnospiraceae	NA
ASV129	46 (0; 76)	0.036717163	0.043294836	Bacteria	Verrucomicrobia	Subdivision5	NA	NA	NA
ASV641	3 (0; 13)	0.039213333	0.046114879	Bacteria	Firmicutes	Clostridia	Clostridiales	Lachnospiraceae	NA
ASV417	11 (2; 36)	0.039690802	0.046552244	Bacteria	Bacteroidetes	Bacteroidia	Bacteroidales	NA	NA

ASV	Difference in location (95% CI)	p-value	BH-FDR p-value	Kingdom	Phylum	Class	Order	Family	Genus
ASV465	6 (0; 47)	0.04029824	0.04713932	Bacteria	Firmicutes	Erysipelotrichia	Erysipelotrichales	Erysipelotrichaceae	NA
ASV688	8.3 (0; 21)	0.040679818	0.047459787	Bacteria	Firmicutes	Clostridia	Clostridiales	Lachnospiraceae	NA
ASV715	4 (0; 15)	0.042283708	0.049200832	Bacteria	Firmicutes	Clostridia	Clostridiales	stridiales_Incertae_Sedis_	Mogibacterium
ASV136	31.2 (0; 99)	0.043598044	0.050596677	Bacteria	Bacteroidetes	NA	NA	NA	NA
ASV271	30 (0; 61)	0.049602895	0.057414374	Bacteria	Bacteroidetes	Bacteroidia	Bacteroidales	NA	NA
ASV616	11 (0; 22)	0.054906103	0.063386365	Bacteria	Firmicutes	Clostridia	Clostridiales	Ruminococcaceae	Sporobacter
ASV532	13 (0; 31)	0.056472363	0.065024314	Bacteria	Firmicutes	Clostridia	Clostridiales	Ruminococcaceae	Ethanoligenens
ASV370	23 (0; 50)	0.057298799	0.06580409	Bacteria	Bacteroidetes	Bacteroidia	Bacteroidales	NA	NA
ASV231	36 (0; 56)	0.059856217	0.068562576	Bacteria	Firmicutes	Clostridia	Clostridiales	Lachnospiraceae	NA
ASV1533	5 (0; 8)	0.061677433	0.070465668	Bacteria	Firmicutes	Clostridia	Clostridiales	Lachnospiraceae	NA
ASV493	11 (-3; 20)	0.065159208	0.07425119	Bacteria	Firmicutes	Clostridia	Clostridiales	Ruminococcaceae	NA
ASV1009	4.5 (0; 13)	0.074765303	0.084978089	Bacteria	Bacteroidetes	Bacteroidia	Bacteroidales	Porphyromonadaceae	NA
ASV906	4.9 (0; 10)	0.077681335	0.088065472	Bacteria	Firmicutes	Clostridia	Clostridiales	NA	NA
ASV226	7 (0; 25)	0.078654156	0.088939699	Bacteria	Bacteroidetes	Flavobacteriia	Flavobacteriales	Flavobacteriaceae	NA
ASV568	4 (0; 26)	0.079437744	0.089596023	Bacteria	Firmicutes	Clostridia	Clostridiales	NA	NA
ASV1715	4 (0; 7)	0.080630165	0.090708936	Bacteria	Firmicutes	Erysipelotrichia	Erysipelotrichales	Erysipelotrichaceae	NA

Abbreviations: ASV, amplicon sequence variant; BH-FDR, Benjamini-Hochberg false discovery rate correction.

Notes: ASVs shown where the BH-FDR p-value < 0.1 for the difference in median location for susceptible versus resistant rhino species as determined by the Wilcoxon rank-sum test.

Supplemental Table 4. ASVs differing in abundance between rhinoceros species.

ASV	p-value	BH-FDR p-value	Kingdom	Phylum	Class	Order	Family	Genus	Species
ASV190	1.91E-63	8.45E-61	Bacteria	Firmicutes	Clostridia	Clostridiales	Ruminococcaceae	Ethanoligenens	NA
ASV6	7.81E-55	1.31E-52	Bacteria	Bacteroidetes	Bacteroidia	Bacteroidales	NA	NA	NA
ASV44	8.89E-55	1.31E-52	Bacteria	Bacteroidetes	Bacteroidia	Bacteroidales	NA	NA	NA
ASV142	1.97E-53	2.18E-51	Bacteria	Firmicutes	Clostridia	Clostridiales	Ruminococcaceae	Sporobacter	NA
ASV29	8.68E-52	7.69E-50	Bacteria	Bacteroidetes	Bacteroidia	Bacteroidales	Prevotellaceae	Prevotella	NA
ASV134	8.73E-47	6.44E-45	Bacteria	Firmicutes	Clostridia	Clostridiales	Lachnospiraceae	NA	NA
ASV17	1.12E-44	7.09E-43	Bacteria	Firmicutes	Clostridia	Clostridiales	Lachnospiraceae	NA	NA
ASV33	1.34E-37	7.44E-36	Bacteria	Firmicutes	Clostridia	Clostridiales	Ruminococcaceae	NA	NA
ASV9	3.82E-37	1.69E-35	Bacteria	Bacteroidetes	NA	NA	NA	NA	NA
ASV76	3.59E-37	1.69E-35	Bacteria	Spirochaetes	Spirochaetia	Spirochaetales	Spirochaetaceae	Treponema	NA
ASV145	4.91E-33	1.98E-31	Bacteria	Firmicutes	Clostridia	Clostridiales	Lachnospiraceae	NA	NA
ASV67	2.54E-31	9.38E-30	Bacteria	Bacteroidetes	Bacteroidia	Bacteroidales	Prevotellaceae	Paraprevotella	NA
ASV94	4.16E-31	1.42E-29	Bacteria	Firmicutes	Clostridia	Clostridiales	Lachnospiraceae	NA	NA
ASV174	1.02E-30	3.24E-29	Bacteria	Firmicutes	Clostridia	Clostridiales	Ruminococcaceae	NA	NA
ASV12	1.24E-30	3.66E-29	Bacteria	Bacteroidetes	Bacteroidia	Bacteroidales	NA	NA	NA
ASV161	1.92E-29	5.32E-28	Bacteria	Bacteroidetes	Bacteroidia	Bacteroidales	Porphyromonadaceae	NA	NA
ASV38	6.34E-29	1.65E-27	Bacteria	Verrucomicrobia	Subdivision5	NA	NA	NA	NA
ASV611	2.18E-28	5.36E-27	Bacteria	Firmicutes	Clostridia	Clostridiales	NA	NA	NA
ASV717	6.38E-26	1.49E-24	Bacteria	Firmicutes	Clostridia	Clostridiales	Ruminococcaceae	NA	NA
ASV206	1.21E-25	2.68E-24	Bacteria	Firmicutes	Clostridia	Clostridiales	Ruminococcaceae	Oscillibacter	NA
ASV351	7.00E-25	1.48E-23	Bacteria	Firmicutes	Clostridia	Clostridiales	Ruminococcaceae	NA	NA
ASV223	8.80E-25	1.77E-23	Bacteria	Bacteroidetes	Bacteroidia	Bacteroidales	Prevotellaceae	Alloprevotella	NA
ASV61	9.36E-25	1.80E-23	Bacteria	Firmicutes	Negativicutes	Selenomonadales	Acidaminococcaceae	Acidaminococcus	NA
ASV251	1.01E-23	1.87E-22	Bacteria	Firmicutes	Clostridia	Clostridiales	Lachnospiraceae	NA	NA
ASV346	2.77E-23	4.87E-22	Bacteria	Firmicutes	Clostridia	Clostridiales	NA	NA	NA
ASV649	2.86E-23	4.87E-22	Bacteria	Firmicutes	Clostridia	Clostridiales	Ruminococcaceae	Sporobacter	NA
ASV256	3.03E-23	4.98E-22	Bacteria	Firmicutes	Clostridia	Clostridiales	NA	NA	NA
ASV283	3.74E-23	5.91E-22	Bacteria	Firmicutes	Clostridia	Clostridiales	Ruminococcaceae	NA	NA
ASV217	1.37E-22	2.09E-21	Bacteria	Bacteroidetes	NA	NA	NA	NA	NA
ASV246	4.82E-22	7.12E-21	Bacteria	Firmicutes	Clostridia	Clostridiales	Ruminococcaceae	NA	NA
ASV707	6.62E-22	9.46E-21	Bacteria	Firmicutes	Clostridia	Clostridiales	Ruminococcaceae	NA	NA
ASV468	2.59E-21	3.58E-20	Bacteria	Bacteroidetes	Bacteroidia	Bacteroidales	NA	NA	NA
ASV661	9.39E-21	1.26E-19	Bacteria	Firmicutes	Clostridia	Clostridiales	Lachnospiraceae	NA	NA
ASV299	1.56E-20	2.04E-19	Bacteria	Firmicutes	Clostridia	Clostridiales	Ruminococcaceae	NA	NA
ASV300	1.66E-20	2.10E-19	Bacteria	Firmicutes	Clostridia	Clostridiales	Lachnospiraceae	NA	NA
ASV258	2.20E-20	2.71E-19	Bacteria	Firmicutes	Erysipelotrichia	Erysipelotrichales	Erysipelotrichaceae	NA	NA
ASV74	2.40E-20	2.87E-19	Bacteria	Bacteroidetes	Bacteroidia	Bacteroidales	NA	NA	NA
ASV712	2.97E-20	3.46E-19	Bacteria	Firmicutes	Clostridia	Clostridiales	NA	NA	NA
ASV284	6.51E-20	7.39E-19	Bacteria	Firmicutes	Clostridia	Clostridiales	Lachnospiraceae	NA	NA
ASV550	1.34E-19	1.49E-18	Bacteria	Firmicutes	Clostridia	Clostridiales	NA	NA	NA
ASV175	1.70E-19	1.84E-18	Bacteria	Bacteroidetes	NA	NA	NA	NA	NA
ASV13	4.42E-19	4.67E-18	Bacteria	Firmicutes	Clostridia	Clostridiales	Ruminococcaceae	Saccharofermentans	NA
ASV539	1.49E-18	1.54E-17	Bacteria	Synergistetes	Synergistia	Synergistales	Synergistaceae	Synergistes	NA
ASV277	2.22E-18	2.23E-17	Bacteria	Firmicutes	Erysipelotrichia	Erysipelotrichales	Erysipelotrichaceae	NA	NA
ASV82	5.16E-18	4.97E-17	Bacteria	Firmicutes	Clostridia	Clostridiales	Lachnospiraceae	Clostridium_XlVa	NA
ASV337	5.06E-18	4.97E-17	Bacteria	Firmicutes	Negativicutes	Selenomonadales	Veillonellaceae	Selenomonas	NA
ASV488	6.16E-18	5.80E-17	Bacteria	Firmicutes	Clostridia	Clostridiales	stridiales_Incertae_Sedis_	Anaerovorax	NA
ASV375	6.69E-18	6.17E-17	Bacteria	Firmicutes	NA	NA	NA	NA	NA
ASV1043	7.27E-18	6.57E-17	Bacteria	Firmicutes	Clostridia	Clostridiales	Lachnospiraceae	NA	NA
ASV116	8.45E-18	7.49E-17	Bacteria	Firmicutes	Clostridia	Clostridiales	Ruminococcaceae	Sporobacter	NA
ASV85	2.40E-17	2.09E-16	Bacteria	Bacteroidetes	Bacteroidia	Bacteroidales	Prevotellaceae	Prevotella	NA

ASV	p-value	BH-FDR p-value	Kingdom	Phylum	Class	Order	Family	Genus	Species
ASV233	7.33E-17	6.25E-16	Bacteria	NA	NA	NA	NA	NA	NA
ASV176	9.57E-17	8.00E-16	Bacteria	Firmicutes	NA	NA	NA	NA	NA
ASV25	2.68E-16	2.18E-15	Bacteria	Firmicutes	Clostridia	Clostridiales	Ruminococcaceae	NA	NA
ASV266	2.70E-16	2.18E-15	Bacteria	Firmicutes	Clostridia	Clostridiales	Syntrophomonadaceae	Pelospora	NA
ASV205	2.77E-16	2.19E-15	Bacteria	NA	NA	NA	NA	NA	NA
ASV481	2.81E-16	2.19E-15	Bacteria	NA	NA	NA	NA	NA	NA
ASV125	3.32E-16	2.54E-15	Bacteria	Firmicutes	Clostridia	Clostridiales	Lachnospiraceae	Clostridium_XlVa	NA
ASV280	3.47E-16	2.61E-15	Bacteria	Bacteroidetes	Bacteroidia	Bacteroidales	Prevotellaceae	NA	NA
ASV793	3.92E-16	2.89E-15	Bacteria	Firmicutes	Clostridia	Clostridiales	Lachnospiraceae	NA	NA
ASV1007	3.97E-16	2.89E-15	Bacteria	NA	NA	NA	NA	NA	NA
ASV354	9.87E-16	7.05E-15	Bacteria	Firmicutes	NA	NA	NA	NA	NA
ASV476	1.10E-15	7.72E-15	Bacteria	Firmicutes	Clostridia	Clostridiales	NA	NA	NA
ASV763	1.27E-15	8.82E-15	Bacteria	Firmicutes	Clostridia	Clostridiales	Ruminococcaceae	NA	NA
ASV489	1.37E-15	9.32E-15	Bacteria	Verrucomicrobia	Subdivision5	NA	NA	NA	NA
ASV606	1.68E-15	1.13E-14	Bacteria	Firmicutes	Clostridia	Clostridiales	Ruminococcaceae	Sporobacter	NA
ASV390	2.81E-15	1.86E-14	Bacteria	Firmicutes	Clostridia	Clostridiales	Ruminococcaceae	Sporobacter	NA
ASV492	4.11E-15	2.68E-14	Bacteria	Bacteroidetes	Bacteroidia	Bacteroidales	NA	NA	NA
ASV197	5.53E-15	3.55E-14	Bacteria	Firmicutes	Clostridia	Clostridiales	Lachnospiraceae	NA	NA
ASV822	6.63E-15	4.20E-14	Bacteria	Firmicutes	Clostridia	Clostridiales	Ruminococcaceae	Sporobacter	NA
ASV124	8.45E-15	5.27E-14	Bacteria	Chloroflexi	Anaerolineae	Anaerolineales	Anaerolineaceae	NA	NA
ASV1151	9.21E-15	5.66E-14	Bacteria	Bacteroidetes	Bacteroidia	Bacteroidales	NA	NA	NA
ASV768	1.08E-14	6.52E-14	Bacteria	Firmicutes	Clostridia	Clostridiales	Ruminococcaceae	NA	NA
ASV240	1.35E-14	8.10E-14	Bacteria	Verrucomicrobia	Subdivision5	NA	NA	NA	NA
ASV321	1.39E-14	8.19E-14	Bacteria	Firmicutes	Clostridia	Clostridiales	Ruminococcaceae	Sporobacter	NA
ASV149	1.63E-14	9.53E-14	Bacteria	Firmicutes	Clostridia	Clostridiales	NA	NA	NA
ASV115	3.94E-14	2.27E-13	Bacteria	Verrucomicrobia	Subdivision5	NA	NA	NA	NA
ASV184	4.69E-14	2.66E-13	Bacteria	Firmicutes	Clostridia	Clostridiales	Lachnospiraceae	Clostridium_XlVa	NA
ASV285	7.03E-14	3.94E-13	Bacteria	Firmicutes	Clostridia	Clostridiales	Ruminococcaceae	Acetivibrio	NA
ASV859	8.00E-14	4.43E-13	Bacteria	Firmicutes	Clostridia	Clostridiales	Ruminococcaceae	Sporobacter	NA
ASV81	9.07E-14	4.96E-13	Bacteria	Bacteroidetes	Bacteroidia	Bacteroidales	Prevotellaceae	Alloprevotella	NA
ASV904	9.89E-14	5.34E-13	Bacteria	Firmicutes	Clostridia	Clostridiales	Ruminococcaceae	Acetivibrio	NA
ASV105	1.01E-13	5.38E-13	Bacteria	Firmicutes	Clostridia	Clostridiales	Ruminococcaceae	NA	NA
ASV304	1.22E-13	6.46E-13	Bacteria	Firmicutes	Clostridia	Clostridiales	Lachnospiraceae	Clostridium_XlVa	NA
ASV733	1.57E-13	8.19E-13	Bacteria	Firmicutes	Clostridia	Clostridiales	NA	NA	NA
ASV65	1.73E-13	8.92E-13	Bacteria	Spirochaetes	Spirochaetia	Spirochaetales	Spirochaetaceae	Treponema	NA
ASV229	2.22E-13	1.13E-12	Bacteria	Firmicutes	Clostridia	Clostridiales	Lachnospiraceae	NA	NA
ASV418	2.63E-13	1.32E-12	Bacteria	NA	NA	NA	NA	NA	NA
ASV40	2.80E-13	1.39E-12	Bacteria	Firmicutes	Clostridia	Clostridiales	Lachnospiraceae	Clostridium_XlVa	NA
ASV1092	4.96E-13	2.44E-12	Bacteria	Firmicutes	Clostridia	Clostridiales	Lachnospiraceae	Blautia	NA
ASV70	5.67E-13	2.76E-12	Bacteria	Firmicutes	Clostridia	Clostridiales	Ruminococcaceae	Sporobacter	NA
ASV21	1.54E-12	7.41E-12	Bacteria	Firmicutes	Clostridia	Clostridiales	Ruminococcaceae	NA	NA
ASV456	2.87E-12	1.36E-11	Bacteria	Firmicutes	Clostridia	Clostridiales	Ruminococcaceae	Sporobacter	NA
ASV745	3.79E-12	1.78E-11	Bacteria	Bacteroidetes	NA	NA	NA	NA	NA
ASV298	8.28E-12	3.86E-11	Bacteria	Firmicutes	Clostridia	Clostridiales	Lachnospiraceae	Clostridium_XlVa	NA
ASV642	9.85E-12	4.55E-11	Bacteria	Bacteroidetes	Bacteroidia	Bacteroidales	Rikenellaceae	Mucinivorans	NA
ASV607	1.25E-11	5.69E-11	Bacteria	Firmicutes	Clostridia	Clostridiales	NA	NA	NA
ASV1020	1.49E-11	6.73E-11	Bacteria	Firmicutes	Clostridia	Clostridiales	Ruminococcaceae	Sporobacter	NA
ASV45	1.57E-11	7.02E-11	Bacteria	Spirochaetes	Spirochaetia	Spirochaetales	Spirochaetaceae	Treponema	NA
ASV366	2.07E-11	9.16E-11	Bacteria	Spirochaetes	Spirochaetia	Spirochaetales	Spirochaetaceae	Treponema	NA
ASV62	2.19E-11	9.61E-11	Bacteria	Bacteroidetes	Bacteroidia	Bacteroidales	Porphyromonadaceae	NA	NA
ASV128	2.37E-11	1.03E-10	Bacteria	Firmicutes	Clostridia	Clostridiales	Ruminococcaceae	Sporobacter	NA
ASV425	3.37E-11	1.45E-10	Bacteria	Firmicutes	NA	NA	NA	NA	NA
ASV767	3.96E-11	1.69E-10	Bacteria	Firmicutes	Clostridia	Clostridiales	Lachnospiraceae	NA	NA

ASV	p-value	BH-FDR p-value	Kingdom	Phylum	Class	Order	Family	Genus	Species
ASV313	5.99E-11	2.53E-10	Bacteria	Firmicutes	Clostridia	Clostridiales	Ruminococcaceae	NA	NA
ASV104	6.57E-11	2.75E-10	Bacteria	Firmicutes	Clostridia	Clostridiales	Ruminococcaceae	Faecalibacterium	NA
ASV727	1.07E-10	4.43E-10	Bacteria	Firmicutes	Clostridia	Clostridiales	Lachnospiraceae	NA	NA
ASV572	1.14E-10	4.68E-10	Bacteria	Armatimonadetes	Armatimonadetes_gp2	NA	NA	NA	NA
ASV980	1.24E-10	5.02E-10	Bacteria	Firmicutes	NA	NA	NA	NA	NA
ASV378	1.46E-10	5.87E-10	Bacteria	Fibrobacteres	Fibrobacteria	Fibrobacterales	Fibrobacteraceae	Fibrobacter	NA
ASV1118	1.50E-10	5.99E-10	Bacteria	Firmicutes	Clostridia	Clostridiales	Lachnospiraceae	NA	NA
ASV806	1.90E-10	7.52E-10	Bacteria	Firmicutes	Clostridia	Clostridiales	NA	NA	NA
ASV55	1.93E-10	7.58E-10	Bacteria	Firmicutes	Clostridia	Clostridiales	Lachnospiraceae	NA	NA
ASV303	2.53E-10	9.84E-10	Bacteria	Firmicutes	Clostridia	Clostridiales	Ruminococcaceae	NA	NA
ASV1359	3.23E-10	1.24E-09	Bacteria	Firmicutes	Clostridia	Clostridiales	Ruminococcaceae	NA	NA
ASV625	3.50E-10	1.33E-09	Bacteria	Firmicutes	Clostridia	Clostridiales	Eubacteriaceae	NA	NA
ASV927	3.52E-10	1.33E-09	Bacteria	Firmicutes	Clostridia	Clostridiales	Lachnospiraceae	NA	NA
ASV51	3.65E-10	1.37E-09	Bacteria	Bacteroidetes	Bacteroidia	Bacteroidales	Porphyromonadaceae	NA	NA
ASV60	5.17E-10	1.92E-09	Bacteria	Verrucomicrobia	Subdivision5	NA	NA	NA	NA
ASV431	6.40E-10	2.36E-09	Bacteria	Firmicutes	Clostridia	Clostridiales	Ruminococcaceae	NA	NA
ASV439	7.61E-10	2.79E-09	Bacteria	Bacteroidetes	Bacteroidia	Bacteroidales	Prevotellaceae	Prevotella	NA
ASV598	7.72E-10	2.80E-09	Bacteria	Firmicutes	Clostridia	Clostridiales	NA	NA	NA
ASV108	8.47E-10	3.05E-09	Bacteria	Firmicutes	Clostridia	Clostridiales	Ruminococcaceae	Sporobacter	NA
ASV52	9.12E-10	3.26E-09	Bacteria	Firmicutes	Clostridia	Clostridiales	Ruminococcaceae	Anaerotruncus	NA
ASV716	1.14E-09	4.03E-09	Bacteria	Firmicutes	Clostridia	Clostridiales	NA	NA	NA
ASV295	1.16E-09	4.07E-09	Bacteria	Firmicutes	NA	NA	NA	NA	NA
ASV706	1.40E-09	4.89E-09	Bacteria	Verrucomicrobia	Subdivision5	NA	NA	NA	NA
ASV19	1.47E-09	5.08E-09	Bacteria	Bacteroidetes	Bacteroidia	Bacteroidales	Porphyromonadaceae	NA	NA
ASV503	2.10E-09	7.22E-09	Bacteria	Firmicutes	Clostridia	Clostridiales	stridiales_Incertae_Sedis_	Anaerovorax	NA
ASV1058	2.25E-09	7.65E-09	Bacteria	Actinobacteria	Actinobacteria	Coriobacteriales	Coriobacteriaceae	NA	NA
ASV1090	2.47E-09	8.37E-09	Bacteria	Firmicutes	Clostridia	Clostridiales	Ruminococcaceae	Sporobacter	NA
ASV956	2.90E-09	9.72E-09	Bacteria	Bacteroidetes	Bacteroidia	Bacteroidales	NA	NA	NA
ASV53	3.33E-09	1.11E-08	Bacteria	Firmicutes	Clostridia	Clostridiales	Ruminococcaceae	NA	NA
ASV583	4.00E-09	1.32E-08	Bacteria	Firmicutes	Clostridia	Clostridiales	stridiales_Incertae_Sedis_	Mogibacterium	NA
ASV75	4.03E-09	1.32E-08	Bacteria	Firmicutes	Clostridia	Clostridiales	Ruminococcaceae	NA	NA
ASV482	4.29E-09	1.40E-08	Bacteria	Proteobacteria	NA	NA	NA	NA	NA
ASV708	4.62E-09	1.49E-08	Bacteria	Planctomycetes	Planctomycetia	Planctomycetales	Planctomycetaceae	NA	NA
ASV164	5.97E-09	1.92E-08	Bacteria	Bacteroidetes	Flavobacteriia	Flavobacteriales	Flavobacteriaceae	NA	NA
ASV199	6.21E-09	1.98E-08	Bacteria	Spirochaetes	Spirochaetia	Spirochaetales	Spirochaetaceae	Treponema	NA
ASV511	7.21E-09	2.28E-08	Bacteria	Firmicutes	Clostridia	Clostridiales	NA	NA	NA
ASV154	7.49E-09	2.35E-08	Bacteria	Firmicutes	NA	NA	NA	NA	NA
ASV120	8.19E-09	2.54E-08	Bacteria	Firmicutes	Clostridia	Clostridiales	NA	NA	NA
ASV1029	8.15E-09	2.54E-08	Bacteria	Firmicutes	Clostridia	Clostridiales	Ruminococcaceae	NA	NA
ASV312	8.85E-09	2.72E-08	Bacteria	Firmicutes	Clostridia	Clostridiales	Lachnospiraceae	NA	NA
ASV103	9.60E-09	2.93E-08	Bacteria	Firmicutes	Clostridia	Clostridiales	Ruminococcaceae	Clostridium_IV	NA
ASV1037	1.13E-08	3.43E-08	Bacteria	Firmicutes	Clostridia	Clostridiales	Ruminococcaceae	Oscillibacter	NA
ASV320	1.19E-08	3.59E-08	Bacteria	NA	NA	NA	NA	NA	NA
ASV372	1.20E-08	3.60E-08	Bacteria	Firmicutes	Clostridia	Clostridiales	Ruminococcaceae	NA	NA
ASV1055	1.80E-08	5.35E-08	Bacteria	Firmicutes	Clostridia	Clostridiales	Ruminococcaceae	NA	NA
ASV657	1.84E-08	5.45E-08	Bacteria	Firmicutes	Clostridia	Clostridiales	Ruminococcaceae	Ruminococcus	NA
ASV362	2.27E-08	6.67E-08	Bacteria	Firmicutes	Clostridia	Clostridiales	Ruminococcaceae	NA	NA
ASV235	2.90E-08	8.44E-08	Bacteria	Firmicutes	Clostridia	Clostridiales	Ruminococcaceae	Intestinimonas	NA
ASV22	3.05E-08	8.83E-08	Bacteria	Bacteroidetes	Bacteroidia	Bacteroidales	Porphyromonadaceae	NA	NA
ASV42	3.11E-08	8.96E-08	Bacteria	Verrucomicrobia	Subdivision5	NA	NA	NA	NA
ASV870	3.40E-08	9.71E-08	Bacteria	Firmicutes	Clostridia	Clostridiales	Lachnospiraceae	Acetitomaculum	NA
ASV263	4.07E-08	1.15E-07	Bacteria	Firmicutes	Negativicutes	Selenomonadales	Veillonellaceae	Selenomonas	NA
ASV34	4.21E-08	1.19E-07	Bacteria	Firmicutes	Clostridia	Clostridiales	Lachnospiraceae	NA	NA

ASV	p-value	BH-FDR p-value	Kingdom	Phylum	Class	Order	Family	Genus	Species
ASV1331	4.97E-08	1.39E-07	Bacteria	Firmicutes	Clostridia	Clostridiales	Lachnospiraceae	NA	NA
ASV267	5.99E-08	1.67E-07	Bacteria	Proteobacteria	Deltaproteobacteria	Desulfovibrionales	NA	NA	NA
ASV738	6.02E-08	1.67E-07	Bacteria	Firmicutes	NA	NA	NA	NA	NA
ASV602	7.20E-08	1.98E-07	Bacteria	Firmicutes	Clostridia	Clostridiales	stridiales_Incertae_Sedis_	Anaerovorax	NA
ASV440	7.41E-08	2.03E-07	Bacteria	Firmicutes	Clostridia	Clostridiales	NA	NA	NA
ASV59	8.01E-08	2.18E-07	Bacteria	Firmicutes	Clostridia	Clostridiales	Lachnospiraceae	NA	NA
ASV1234	9.21E-08	2.49E-07	Bacteria	NA	NA	NA	NA	NA	NA
ASV746	1.05E-07	2.81E-07	Bacteria	Verrucomicrobia	Subdivision5	NA	NA	NA	NA
ASV293	1.09E-07	2.90E-07	Bacteria	Firmicutes	Clostridia	Clostridiales	Ruminococcaceae	Acetivibrio	NA
ASV413	1.17E-07	3.12E-07	Bacteria	Chloroflexi	Anaerolineae	Anaerolineales	Anaerolineaceae	Ornatilinea	NA
ASV988	1.33E-07	3.50E-07	Bacteria	Bacteroidetes	Bacteroidia	Bacteroidales	Marinilabiliaceae	NA	NA
ASV724	1.44E-07	3.79E-07	Bacteria	Firmicutes	Clostridia	Clostridiales	NA	NA	NA
ASV739	1.48E-07	3.86E-07	Bacteria	Firmicutes	Clostridia	Clostridiales	Ruminococcaceae	NA	NA
ASV83	1.58E-07	4.08E-07	Bacteria	Bacteroidetes	Bacteroidia	Bacteroidales	Prevotellaceae	NA	NA
ASV368	1.70E-07	4.39E-07	Bacteria	Firmicutes	Clostridia	Clostridiales	NA	NA	NA
ASV126	2.44E-07	6.24E-07	Bacteria	Firmicutes	Clostridia	Clostridiales	Ruminococcaceae	Sporobacter	NA
ASV297	2.72E-07	6.92E-07	Bacteria	Firmicutes	Clostridia	Clostridiales	Lachnospiraceae	NA	NA
ASV170	2.76E-07	7.00E-07	Bacteria	Firmicutes	Negativicutes	Selenomonadales	Veillonellaceae	Selenomonas	NA
ASV306	2.82E-07	7.09E-07	Bacteria	Firmicutes	Clostridia	Clostridiales	Ruminococcaceae	NA	NA
ASV713	3.32E-07	8.31E-07	Bacteria	Firmicutes	Clostridia	Clostridiales	stridiales_Incertae_Sedis_	Anaerovorax	NA
ASV1103	3.51E-07	8.74E-07	Bacteria	Firmicutes	Clostridia	Clostridiales	NA	NA	NA
ASV123	3.77E-07	9.34E-07	Bacteria	Bacteroidetes	NA	NA	NA	NA	NA
ASV827	4.14E-07	1.02E-06	Bacteria	Firmicutes	Clostridia	Clostridiales	Ruminococcaceae	NA	NA
ASV71	4.19E-07	1.02E-06	Bacteria	Firmicutes	Clostridia	Clostridiales	Lachnospiraceae	Clostridium_XlVa	NA
ASV1013	4.35E-07	1.06E-06	Bacteria	Bacteroidetes	Bacteroidia	Bacteroidales	Prevotellaceae	Prevotella	NA
ASV1423	4.36E-07	1.06E-06	Bacteria	Tenericutes	Mollicutes	Anaeroplasmatales	Anaeroplasmataceae	Anaeroplasma	NA
ASV1061	4.52E-07	1.09E-06	Bacteria	Firmicutes	Clostridia	Clostridiales	NA	NA	NA
ASV31	5.23E-07	1.25E-06	Bacteria	Firmicutes	Clostridia	Clostridiales	Lachnospiraceae	NA	NA
ASV915	5.37E-07	1.28E-06	Bacteria	Firmicutes	Erysipelotrichia	Erysipelotrichales	Erysipelotrichaceae	Holdemania	NA
ASV994	5.51E-07	1.30E-06	Bacteria	NA	NA	NA	NA	NA	NA
ASV846	6.14E-07	1.45E-06	Bacteria	Bacteroidetes	NA	NA	NA	NA	NA
ASV545	6.36E-07	1.49E-06	Bacteria	Firmicutes	Clostridia	Clostridiales	Ruminococcaceae	NA	NA
ASV86	6.85E-07	1.60E-06	Bacteria	Bacteroidetes	NA	NA	NA	NA	NA
ASV641	7.94E-07	1.84E-06	Bacteria	Firmicutes	Clostridia	Clostridiales	Lachnospiraceae	NA	NA
ASV635	8.29E-07	1.91E-06	Bacteria	Firmicutes	Clostridia	Clostridiales	NA	NA	NA
ASV376	8.72E-07	2.00E-06	Bacteria	Bacteroidetes	Bacteroidia	Bacteroidales	NA	NA	NA
ASV339	9.62E-07	2.20E-06	Bacteria	Firmicutes	Clostridia	Clostridiales	stridiales_Incertae_Sedis_	Anaerovorax	NA
ASV1202	9.76E-07	2.22E-06	Bacteria	Firmicutes	Clostridia	Clostridiales	NA	NA	NA
ASV510	1.14E-06	2.58E-06	Bacteria	Firmicutes	Clostridia	Clostridiales	Clostridiaceae_1	NA	NA
ASV429	1.19E-06	2.67E-06	Bacteria	Firmicutes	Clostridia	Clostridiales	Lachnospiraceae	NA	NA
ASV646	1.44E-06	3.22E-06	Bacteria	Firmicutes	Clostridia	Clostridiales	Lachnospiraceae	NA	NA
ASV680	1.85E-06	4.13E-06	Bacteria	Firmicutes	Clostridia	Clostridiales	Lachnospiraceae	Acetatifactor	NA
ASV35	2.04E-06	4.51E-06	Bacteria	Fibrobacteres	Fibrobacteria	Fibrobacterales	Fibrobacteraceae	Fibrobacter	NA
ASV853	2.17E-06	4.79E-06	Bacteria	Firmicutes	Clostridia	Clostridiales	Lachnospiraceae	NA	NA
ASV91	2.25E-06	4.94E-06	Bacteria	Firmicutes	Clostridia	Clostridiales	NA	NA	NA
ASV144	2.44E-06	5.33E-06	Bacteria	Firmicutes	Clostridia	Clostridiales	NA	NA	NA
ASV1004	2.55E-06	5.54E-06	Bacteria	Bacteroidetes	NA	NA	NA	NA	NA
ASV131	2.62E-06	5.66E-06	Bacteria	Firmicutes	Clostridia	Clostridiales	Ruminococcaceae	NA	NA
ASV84	2.76E-06	5.93E-06	Bacteria	Verrucomicrobia	Subdivision5	NA	NA	NA	NA
ASV243	2.80E-06	5.99E-06	Bacteria	Spirochaetes	Spirochaetia	Spirochaetales	Spirochaetaceae	Treponema	NA
ASV839	3.22E-06	6.87E-06	Bacteria	Firmicutes	Clostridia	Clostridiales	NA	NA	NA
ASV46	3.42E-06	7.24E-06	Bacteria	Firmicutes	Clostridia	Clostridiales	Ruminococcaceae	Oscillibacter	NA
ASV1335	3.51E-06	7.41E-06	Bacteria	Firmicutes	Clostridia	Clostridiales	Lachnospiraceae	NA	NA

ASV	p-value	BH-FDR p-value	Kingdom	Phylum	Class	Order	Family	Genus	Species
ASV1071	3.66E-06	7.69E-06	Bacteria	Bacteroidetes	Bacteroidia	Bacteroidales	NA	NA	NA
ASV386	3.71E-06	7.76E-06	Bacteria	Chloroflexi	Anaerolineae	Anaerolineales	Anaerolineaceae	NA	NA
ASV417	4.65E-06	9.68E-06	Bacteria	Bacteroidetes	Bacteroidia	Bacteroidales	NA	NA	NA
ASV567	5.07E-06	1.05E-05	Bacteria	Firmicutes	Clostridia	Clostridiales	Ruminococcaceae	NA	NA
ASV433	5.11E-06	1.05E-05	Bacteria	Verrucomicrobia	Subdivision5	NA	NA	NA	NA
ASV803	5.16E-06	1.06E-05	Bacteria	Firmicutes	Clostridia	Clostridiales	NA	NA	NA
ASV715	5.34E-06	1.09E-05	Bacteria	Firmicutes	Clostridia	Clostridiales	stridiales_Incertae_Sedis_	Mogibacterium	NA
ASV574	5.69E-06	1.16E-05	Bacteria	Firmicutes	Clostridia	Clostridiales	NA	NA	NA
ASV423	5.77E-06	1.17E-05	Bacteria	Firmicutes	Clostridia	Clostridiales	Ruminococcaceae	NA	NA
ASV1432	5.93E-06	1.19E-05	Bacteria	Bacteroidetes	Bacteroidia	Bacteroidales	Rikenellaceae	Mucinivorans	NA
ASV324	6.43E-06	1.29E-05	Bacteria	Actinobacteria	Actinobacteria	Coriobacteriales	Coriobacteriaceae	NA	NA
ASV1262	6.67E-06	1.33E-05	Bacteria	Firmicutes	Clostridia	Clostridiales	NA	NA	NA
ASV328	6.70E-06	1.33E-05	Bacteria	NA	NA	NA	NA	NA	NA
ASV160	7.30E-06	1.44E-05	Bacteria	Verrucomicrobia	Subdivision5	NA	NA	NA	NA
ASV981	7.49E-06	1.47E-05	Bacteria	Proteobacteria	Deltaproteobacteria	NA	NA	NA	NA
ASV63	7.98E-06	1.56E-05	Bacteria	Firmicutes	NA	NA	NA	NA	NA
ASV1273	9.92E-06	1.94E-05	Bacteria	Firmicutes	NA	NA	NA	NA	NA
ASV381	1.02E-05	1.99E-05	Bacteria	Bacteroidetes	NA	NA	NA	NA	NA
ASV703	1.06E-05	2.05E-05	Bacteria	Firmicutes	Clostridia	Clostridiales	NA	NA	NA
ASV2053	1.12E-05	2.15E-05	Bacteria	Firmicutes	NA	NA	NA	NA	NA
ASV2087	1.12E-05	2.15E-05	Bacteria	Firmicutes	Clostridia	Clostridiales	Lachnospiraceae	Anaerosporobacter	NA
ASV1999	1.14E-05	2.17E-05	Bacteria	Actinobacteria	Actinobacteria	Coriobacteriales	Coriobacteriaceae	NA	NA
ASV1	1.17E-05	2.23E-05	Bacteria	Firmicutes	Clostridia	Clostridiales	Ruminococcaceae	Ruminococcus	NA
ASV99	1.27E-05	2.41E-05	Bacteria	Firmicutes	Clostridia	Clostridiales	Lachnospiraceae	NA	NA
ASV461	1.75E-05	3.29E-05	Bacteria	Firmicutes	Bacilli	Lactobacillales	Lactobacillaceae	Lactobacillus	NA
ASV16	1.80E-05	3.37E-05	Bacteria	Bacteroidetes	NA	NA	NA	NA	NA
ASV2060	2.04E-05	3.82E-05	Bacteria	Firmicutes	Clostridia	Clostridiales	ostridiales_Incertae_Sedis_	Clostridium_XII	NA
ASV2	2.18E-05	4.05E-05	Bacteria	Firmicutes	NA	NA	NA	NA	NA
ASV191	2.26E-05	4.18E-05	Bacteria	Spirochaetes	Spirochaetia	Spirochaetales	Spirochaetaceae	Treponema	NA
ASV109	2.27E-05	4.18E-05	Bacteria	Firmicutes	Clostridia	Clostridiales	Ruminococcaceae	Clostridium_IV	NA
ASV419	2.36E-05	4.34E-05	Bacteria	Verrucomicrobia	Subdivision5	NA	NA	NA	NA
ASV250	2.38E-05	4.36E-05	Bacteria	Firmicutes	Clostridia	Clostridiales	Lachnospiraceae	NA	NA
ASV1578	2.53E-05	4.61E-05	Bacteria	Firmicutes	Clostridia	Clostridiales	Ruminococcaceae	NA	NA
ASV1761	2.64E-05	4.79E-05	Bacteria	Firmicutes	Clostridia	Clostridiales	Lachnospiraceae	NA	NA
ASV742	2.80E-05	5.06E-05	Bacteria	Firmicutes	Clostridia	Clostridiales	NA	NA	NA
ASV801	2.88E-05	5.19E-05	Bacteria	Firmicutes	Clostridia	Clostridiales	Ruminococcaceae	Clostridium_IV	NA
ASV847	3.18E-05	5.69E-05	Bacteria	Bacteroidetes	NA	NA	NA	NA	NA
ASV950	3.32E-05	5.92E-05	Bacteria	Bacteroidetes	Bacteroidia	Bacteroidales	NA	NA	NA
ASV654	3.36E-05	5.98E-05	Bacteria	Firmicutes	Clostridia	Clostridiales	Lachnospiraceae	NA	NA
ASV392	3.49E-05	6.19E-05	Bacteria	Firmicutes	Clostridia	Clostridiales	Ruminococcaceae	Acetivibrio	NA
ASV1279	3.86E-05	6.82E-05	Bacteria	Firmicutes	Clostridia	Clostridiales	NA	NA	NA
ASV1124	4.15E-05	7.30E-05	Bacteria	Firmicutes	Clostridia	Clostridiales	Lachnospiraceae	NA	NA
ASV428	5.07E-05	8.88E-05	Bacteria	Firmicutes	Clostridia	Clostridiales	Ruminococcaceae	Sporobacter	NA
ASV943	5.39E-05	9.40E-05	Bacteria	Firmicutes	Clostridia	Clostridiales	Ruminococcaceae	Oscillibacter	NA
ASV1813	5.51E-05	9.56E-05	Bacteria	Bacteroidetes	Bacteroidia	Bacteroidales	Bacteroidaceae	Anaerorhabdus	NA
ASV519	6.06E-05	0.00010494	Bacteria	Firmicutes	NA	NA	NA	NA	NA
ASV432	6.53E-05	0.00011234	Bacteria	Firmicutes	Clostridia	Clostridiales	NA	NA	NA
ASV858	6.54E-05	0.00011234	Bacteria	Firmicutes	Clostridia	Clostridiales	stridiales_Incertae_Sedis_	NA	NA
ASV890	6.64E-05	0.000113601	Bacteria	Firmicutes	Clostridia	Clostridiales	Ruminococcaceae	NA	NA
ASV310	6.82E-05	0.000116205	Bacteria	Firmicutes	Clostridia	Clostridiales	Lachnospiraceae	NA	NA
ASV678	7.16E-05	0.000121468	Bacteria	Lentisphaerae	Oligosphaeria	Oligosphaerales	Oligosphaeraceae	Oligosphaera	NA
ASV1297	7.24E-05	0.000122472	Bacteria	Firmicutes	Clostridia	Clostridiales	Ruminococcaceae	NA	NA
ASV1520	7.48E-05	0.000126022	Bacteria	Firmicutes	Clostridia	Clostridiales	NA	NA	NA

ASV	p-value	BH-FDR p-value	Kingdom	Phylum	Class	Order	Family	Genus	Species
ASV112	7.54E-05	0.000126536	Bacteria	Firmicutes	Clostridia	Clostridiales	NA	NA	NA
ASV673	7.57E-05	0.000126569	Bacteria	Firmicutes	Clostridia	Clostridiales	Ruminococcaceae	NA	NA
ASV1325	7.73E-05	0.00012869	Bacteria	Firmicutes	Clostridia	Clostridiales	NA	NA	NA
ASV509	7.81E-05	0.000129534	Bacteria	Firmicutes	NA	NA	NA	NA	NA
ASV192	7.88E-05	0.000130201	Bacteria	Firmicutes	Clostridia	Clostridiales	Ruminococcaceae	NA	NA
ASV908	8.18E-05	0.000134737	Bacteria	Firmicutes	Clostridia	Clostridiales	Lachnospiraceae	NA	NA
ASV32	8.25E-05	0.000135435	Bacteria	Firmicutes	Clostridia	Clostridiales	Ruminococcaceae	NA	NA
ASV942	8.58E-05	0.000140222	Bacteria	Firmicutes	Clostridia	Clostridiales	Ruminococcaceae	Sporobacter	NA
ASV648	8.75E-05	0.000142546	Bacteria	Verrucomicrobia	Subdivision5	NA	NA	NA	NA
ASV66	9.36E-05	0.000151917	Bacteria	Firmicutes	Clostridia	Clostridiales	Lachnospiraceae	NA	NA
ASV335	0.000101303	0.00016342	Bacteria	Firmicutes	Clostridia	Clostridiales	Ruminococcaceae	Saccharofermentans	NA
ASV758	0.000101446	0.00016342	Bacteria	Firmicutes	Clostridia	Clostridiales	Lachnospiraceae	NA	NA
ASV1077	0.000106078	0.000170262	Bacteria	Firmicutes	Clostridia	Clostridiales	NA	NA	NA
ASV466	0.000107212	0.000171461	Bacteria	Firmicutes	Clostridia	Clostridiales	Ruminococcaceae	NA	NA
ASV1377	0.000110919	0.000176753	Bacteria	Firmicutes	Clostridia	Clostridiales	Lachnospiraceae	Acetatifactor	NA
ASV578	0.000115854	0.000183955	Bacteria	Firmicutes	Erysipelotrichia	Erysipelotrichales	Erysipelotrichaceae	NA	NA
ASV1426	0.000119712	0.000189401	Bacteria	Firmicutes	Clostridia	NA	NA	NA	NA
ASV499	0.000124724	0.000196629	Bacteria	Firmicutes	Clostridia	Clostridiales	Ruminococcaceae	NA	NA
ASV968	0.000126167	0.000197593	Bacteria	Firmicutes	Clostridia	Clostridiales	Ruminococcaceae	NA	NA
ASV1009	0.000126228	0.000197593	Bacteria	Bacteroidetes	Bacteroidia	Bacteroidales	Porphyromonadaceae	NA	NA
ASV886	0.000146489	0.000228503	Bacteria	Bacteroidetes	Bacteroidia	Bacteroidales	Prevotellaceae	Paraprevotella	NA
ASV216	0.000161242	0.000250632	Bacteria	Bacteroidetes	NA	NA	NA	NA	NA
ASV1703	0.000164479	0.00025477	Bacteria	Firmicutes	Clostridia	Clostridiales	Lachnospiraceae	NA	NA
ASV1102	0.000169184	0.000261144	Bacteria	Actinobacteria	Actinobacteria	Coriobacteriales	Coriobacteriaceae	Parvibacter	NA
ASV1094	0.00017115	0.000263262	Bacteria	Firmicutes	Clostridia	Clostridiales	Ruminococcaceae	NA	NA
ASV522	0.000172202	0.000263964	Bacteria	Verrucomicrobia	Subdivision5	NA	NA	NA	NA
ASV359	0.000175086	0.00026746	Bacteria	Firmicutes	Clostridia	Clostridiales	Ruminococcaceae	NA	NA
ASV494	0.000179314	0.000272976	Bacteria	Firmicutes	Clostridia	Clostridiales	Lachnospiraceae	NA	NA
ASV1160	0.000181647	0.000275581	Bacteria	Firmicutes	Clostridia	Clostridiales	Lachnospiraceae	NA	NA
ASV1634	0.00018297	0.000276641	Bacteria	Firmicutes	Clostridia	Clostridiales	NA	NA	NA
ASV454	0.000186093	0.000280405	Bacteria	Firmicutes	Clostridia	Clostridiales	Lachnospiraceae	NA	NA
ASV383	0.000189645	0.00028479	Bacteria	Verrucomicrobia	Subdivision5	NA	NA	NA	NA
ASV925	0.000200324	0.000299809	Bacteria	Bacteroidetes	Bacteroidia	Bacteroidales	Rikenellaceae	Mucinivorans	NA
ASV441	0.000201128	0.000299999	Bacteria	Firmicutes	Clostridia	Clostridiales	Ruminococcaceae	Sporobacter	NA
ASV553	0.000204921	0.000304631	Bacteria	Firmicutes	Clostridia	Clostridiales	Lachnospiraceae	NA	NA
ASV636	0.000245317	0.000363463	Bacteria	Firmicutes	Clostridia	Clostridiales	Ruminococcaceae	Sporobacter	NA
ASV1950	0.000263191	0.000388646	Bacteria	Firmicutes	Clostridia	Clostridiales	Clostridiaceae_1	NA	NA
ASV1536	0.000292982	0.0004312	Bacteria	Firmicutes	Clostridia	Clostridiales	Ruminococcaceae	NA	NA
ASV1248	0.000306141	0.000449074	Bacteria	Chloroflexi	Anaerolineae	Anaerolineales	Anaerolineaceae	Ornatilinea	NA
ASV959	0.000311272	0.000455094	Bacteria	Firmicutes	Clostridia	Clostridiales	stridiales_Incertae_Sedis_	Anaerovorax	NA
ASV1666	0.00031322	0.000456436	Bacteria	Firmicutes	Clostridia	Clostridiales	Ruminococcaceae	NA	NA
ASV593	0.000402358	0.000584409	Bacteria	Firmicutes	Clostridia	Clostridiales	Lachnospiraceae	NA	NA
ASV398	0.000424879	0.000615102	Bacteria	Bacteroidetes	Bacteroidia	Bacteroidales	Prevotellaceae	Prevotella	NA
ASV1555	0.000437638	0.00063151	Bacteria	Firmicutes	Clostridia	Clostridiales	NA	NA	NA
ASV918	0.000453214	0.000651862	Bacteria	Firmicutes	Clostridia	Clostridiales	Ruminococcaceae	Sporobacter	NA
ASV1495	0.000467054	0.000669595	Bacteria	Firmicutes	Clostridia	Clostridiales	Ruminococcaceae	NA	NA
ASV480	0.000495343	0.000705585	Bacteria	Verrucomicrobia	Subdivision5	NA	NA	NA	NA
ASV2015	0.000493824	0.000705585	Bacteria	Actinobacteria	Actinobacteria	Coriobacteriales	Coriobacteriaceae	NA	NA
ASV427	0.000512165	0.000727208	Bacteria	Firmicutes	Clostridia	Clostridiales	Lachnospiraceae	NA	NA
ASV1057	0.000530471	0.000750794	Bacteria	Planctomycetes	Planctomycetia	Planctomycetales	Planctomycetaceae	Pirellula	NA
ASV655	0.000561791	0.000792591	Bacteria	Firmicutes	Clostridia	Clostridiales	Lachnospiraceae	Lachnobacterium	NA
ASV1873	0.000563765	0.000792851	Bacteria	NA	NA	NA	NA	NA	NA
ASV97	0.000575111	0.000806248	Bacteria	Spirochaetes	Spirochaetia	Spirochaetales	Spirochaetaceae	Treponema	NA

ASV	p-value	BH-FDR p-value	Kingdom	Phylum	Class	Order	Family	Genus	Species
ASV225	0.000583825	0.000815882	Bacteria	Firmicutes	Clostridia	Clostridiales	stridiales_Incertae_Sedis_	Anaerovorax	NA
ASV449	0.000590688	0.000822876	Bacteria	Firmicutes	Clostridia	Clostridiales	Ruminococcaceae	NA	NA
ASV518	0.000626056	0.000867872	Bacteria	Firmicutes	NA	NA	NA	NA	NA
ASV576	0.000626905	0.000867872	Bacteria	Firmicutes	Clostridia	Clostridiales	Lachnospiraceae	NA	NA
ASV669	0.000695679	0.00096008	Bacteria	Firmicutes	Clostridia	Clostridiales	Lachnospiraceae	NA	NA
ASV618	0.00070728	0.000973059	Bacteria	Bacteroidetes	Bacteroidia	Bacteroidales	Prevotellaceae	Prevotella	NA
ASV855	0.000722451	0.000990854	Bacteria	Firmicutes	Erysipelotrichia	Erysipelotrichales	Erysipelotrichaceae	NA	NA
ASV515	0.000748044	0.001019642	Bacteria	Bacteroidetes	Bacteroidia	Bacteroidales	Porphyromonadaceae	NA	NA
ASV1871	0.000746374	0.001019642	Bacteria	Firmicutes	Clostridia	Clostridiales	NA	NA	NA
ASV101	0.000771611	0.001048539	Bacteria	Firmicutes	Clostridia	Clostridiales	Lachnospiraceae	NA	NA
ASV500	0.000837764	0.001134952	Bacteria	Firmicutes	Clostridia	Clostridiales	Lachnospiraceae	NA	NA
ASV2090	0.000865449	0.001168884	Bacteria	Firmicutes	Erysipelotrichia	Erysipelotrichales	Erysipelotrichaceae	NA	NA
ASV396	0.000874696	0.001177782	Bacteria	Firmicutes	Clostridia	Clostridiales	NA	NA	NA
ASV590	0.000922759	0.001238735	Bacteria	Firmicutes	Clostridia	Clostridiales	Lachnospiraceae	NA	NA
ASV1567	0.0009389	0.001256595	Bacteria	Firmicutes	Clostridia	Clostridiales	NA	NA	NA
ASV1230	0.001107732	0.001478089	Bacteria	Proteobacteria	Gammaproteobacteria	NA	NA	NA	NA
ASV173	0.001153772	0.001534898	Bacteria	Bacteroidetes	NA	NA	NA	NA	NA
ASV1632	0.001157382	0.001535091	Bacteria	Firmicutes	Clostridia	Clostridiales	NA	NA	NA
ASV917	0.001233675	0.001631398	Bacteria	Bacteroidetes	Bacteroidia	Bacteroidales	NA	NA	NA
ASV1018	0.001275197	0.001681286	Bacteria	Firmicutes	Clostridia	Clostridiales	Ruminococcaceae	NA	NA
ASV581	0.001285171	0.001689409	Bacteria	Firmicutes	Clostridia	Clostridiales	Ruminococcaceae	NA	NA
ASV1123	0.001289786	0.00169046	Bacteria	Bacteroidetes	Bacteroidia	Bacteroidales	NA	NA	NA
ASV465	0.00130014	0.001699004	Bacteria	Firmicutes	Erysipelotrichia	Erysipelotrichales	Erysipelotrichaceae	NA	NA
ASV404	0.001423912	0.001855274	Bacteria	Bacteroidetes	NA	NA	NA	NA	NA
ASV1326	0.001474478	0.001915524	Bacteria	Firmicutes	Clostridia	Clostridiales	Ruminococcaceae	NA	NA
ASV182	0.001623219	0.00210259	Bacteria	Firmicutes	Clostridia	Clostridiales	Lachnospiraceae	NA	NA
ASV1211	0.001736959	0.002243361	Bacteria	Firmicutes	Clostridia	Clostridiales	Lachnospiraceae	NA	NA
ASV407	0.001808188	0.002328567	Bacteria	Verrucomicrobia	Subdivision5	NA	NA	NA	NA
ASV923	0.001876813	0.002409936	Bacteria	Firmicutes	Clostridia	Clostridiales	Ruminococcaceae	NA	NA
ASV989	0.002004834	0.002566883	Bacteria	Firmicutes	Clostridia	Clostridiales	Ruminococcaceae	NA	NA
ASV1397	0.002264544	0.002891046	Bacteria	Firmicutes	Clostridia	Clostridiales	NA	NA	NA
ASV1420	0.00228698	0.002911299	Bacteria	Firmicutes	Clostridia	Clostridiales	Ruminococcaceae	Intestinimonas	NA
ASV402	0.002306266	0.002927439	Bacteria	Firmicutes	NA	NA	NA	NA	NA
ASV1379	0.002538932	0.003213563	Bacteria	Firmicutes	Clostridia	Clostridiales	stridiales_Incertae_Sedis_	Anaerovorax	NA
ASV1245	0.002559501	0.003230368	Bacteria	Actinobacteria	Actinobacteria	Coriobacteriales	Coriobacteriaceae	Parvibacter	NA
ASV2097	0.002617249	0.003293867	Bacteria	Proteobacteria	NA	NA	NA	NA	NA
ASV1719	0.002677132	0.003359687	Bacteria	Firmicutes	Clostridia	Clostridiales	NA	NA	NA
ASV238	0.00316051	0.003955101	Bacteria	Firmicutes	Clostridia	Clostridiales	Ruminococcaceae	Sporobacter	NA
ASV309	0.003374852	0.004211435	Bacteria	Firmicutes	Clostridia	Clostridiales	NA	NA	NA
ASV1421	0.003457701	0.004302701	Bacteria	Firmicutes	Clostridia	Clostridiales	Ruminococcaceae	Clostridium_IV	NA
ASV910	0.003526904	0.004376523	Bacteria	Firmicutes	Clostridia	Clostridiales	NA	NA	NA
ASV1238	0.004099572	0.005072935	Bacteria	Firmicutes	Clostridia	Clostridiales	Lachnospiraceae	Clostridium_XlVb	NA
ASV906	0.004261404	0.005258501	Bacteria	Firmicutes	Clostridia	Clostridiales	NA	NA	NA
ASV637	0.004493936	0.005530038	Bacteria	Actinobacteria	Actinobacteria	Coriobacteriales	Coriobacteriaceae	NA	NA
ASV129	0.004742717	0.00582001	Bacteria	Verrucomicrobia	Subdivision5	NA	NA	NA	NA
ASV2021	0.004904015	0.006001322	Bacteria	Firmicutes	Clostridia	Clostridiales	Ruminococcaceae	NA	NA
ASV1226	0.005497414	0.006708965	Bacteria	Verrucomicrobia	Subdivision5	NA	NA	NA	NA
ASV467	0.005556905	0.006762936	Bacteria	Firmicutes	Clostridia	Clostridiales	Ruminococcaceae	NA	NA
ASV645	0.005997233	0.007278834	Bacteria	Firmicutes	Clostridia	Clostridiales	stridiales_Incertae_Sedis_	Anaerovorax	NA
ASV562	0.00606144	0.00733666	Bacteria	Firmicutes	Clostridia	Clostridiales	NA	NA	NA
ASV1178	0.006084902	0.007344991	Bacteria	Firmicutes	Clostridia	Clostridiales	NA	NA	NA
ASV2009	0.006492197	0.007815335	Bacteria	Firmicutes	Erysipelotrichia	Erysipelotrichales	Erysipelotrichaceae	NA	NA
ASV210	0.007703108	0.009222911	Bacteria	Firmicutes	Clostridia	Clostridiales	Ruminococcaceae	NA	NA

ASV	p-value	BH-FDR p-value	Kingdom	Phylum	Class	Order	Family	Genus	Species
ASV236	0.007689732	0.009222911	Bacteria	NA	NA	NA	NA	NA	NA
ASV2387	0.008684191	0.010369533	Bacteria	Firmicutes	Clostridia	Clostridiales	Ruminococcaceae	Papillibacter	NA
ASV1831	0.008716845	0.010380544	Bacteria	Firmicutes	Clostridia	Clostridiales	Ruminococcaceae	Sporobacter	NA
ASV805	0.008793737	0.010444036	Bacteria	Firmicutes	Clostridia	Clostridiales	Lachnospiraceae	Clostridium_XlVa	NA
ASV538	0.009473516	0.011221303	Bacteria	Firmicutes	Clostridia	Clostridiales	Lachnospiraceae	Roseburia	NA
ASV595	0.00977765	0.011550664	Bacteria	Firmicutes	Clostridia	Clostridiales	NA	NA	NA
ASV370	0.010483861	0.012351995	Bacteria	Bacteroidetes	Bacteroidia	Bacteroidales	NA	NA	NA
ASV106	0.011342901	0.01332866	Bacteria	Firmicutes	Clostridia	Clostridiales	Ruminococcaceae	NA	NA
ASV389	0.011396448	0.013356155	Bacteria	Bacteroidetes	NA	NA	NA	NA	NA
ASV1552	0.012822727	0.014988043	Bacteria	Firmicutes	NA	NA	NA	NA	NA
ASV136	0.013109193	0.01528256	Bacteria	Bacteroidetes	NA	NA	NA	NA	NA
ASV177	0.014838903	0.017253633	Bacteria	Firmicutes	Clostridia	Clostridiales	Ruminococcaceae	Intestinimonas	NA
ASV271	0.014942485	0.017328588	Bacteria	Bacteroidetes	Bacteroidia	Bacteroidales	NA	NA	NA
ASV1190	0.016101791	0.018624265	Bacteria	Actinobacteria	Actinobacteria	Coriobacteriales	Coriobacteriaceae	Parvibacter	NA
ASV936	0.018580731	0.021435583	Bacteria	Firmicutes	Clostridia	Clostridiales	Ruminococcaceae	NA	NA
ASV566	0.018981775	0.021841367	Bacteria	Verrucomicrobia	Subdivision5	NA	NA	NA	NA
ASV326	0.019186853	0.022020144	Bacteria	Firmicutes	Clostridia	Clostridiales	Lachnospiraceae	Pseudobutyrivibrio	NA
ASV493	0.020383477	0.023333024	Bacteria	Firmicutes	Clostridia	Clostridiales	Ruminococcaceae	NA	NA
ASV868	0.020700895	0.023635301	Bacteria	Firmicutes	Clostridia	Clostridiales	Lachnospiraceae	NA	NA
ASV231	0.022352088	0.025454949	Bacteria	Firmicutes	Clostridia	Clostridiales	Lachnospiraceae	NA	NA
ASV1562	0.024311186	0.027615014	Bacteria	Firmicutes	Clostridia	Clostridiales	Ruminococcaceae	Sporobacter	NA
ASV700	0.025733702	0.029156087	Bacteria	Firmicutes	Clostridia	NA	NA	NA	NA
ASV1422	0.026645385	0.030112004	Bacteria	Firmicutes	Clostridia	Clostridiales	Lachnospiraceae	NA	NA
ASV1249	0.02832129	0.031924508	Bacteria	Firmicutes	Clostridia	Clostridiales	Lachnospiraceae	NA	NA
ASV736	0.032830314	0.036913272	Bacteria	Firmicutes	Negativicutes	Selenomonadales	Veillonellaceae	NA	NA
ASV24	0.035810494	0.040162149	Bacteria	Firmicutes	Clostridia	Clostridiales	Ruminococcaceae	NA	NA
ASV879	0.037254679	0.04167632	Bacteria	Firmicutes	Clostridia	Clostridiales	Ruminococcaceae	NA	NA
ASV1350	0.037486265	0.041829761	Bacteria	Cyanobacteria/Chloroplast	Chloroplast	Chloroplast	Streptophyta	NA	NA
ASV536	0.037886893	0.042170587	Bacteria	Firmicutes	Erysipelotrichia	Erysipelotrichales	Erysipelotrichaceae	NA	NA
ASV1281	0.039209978	0.043533886	Bacteria	Firmicutes	Erysipelotrichia	Erysipelotrichales	Erysipelotrichaceae	Bulleidia	NA
ASV947	0.041735256	0.046221796	Bacteria	Firmicutes	Clostridia	Clostridiales	Lachnospiraceae	NA	NA
ASV952	0.042017644	0.046418495	Bacteria	Firmicutes	Clostridia	Clostridiales	NA	NA	NA
ASV1486	0.046018309	0.050711719	Bacteria	Firmicutes	Clostridia	Clostridiales	Ruminococcaceae	Sporobacter	NA
ASV564	0.046770709	0.051412963	Bacteria	Firmicutes	Clostridia	Clostridiales	Lachnospiraceae	NA	NA
ASV287	0.054572151	0.059692501	Bacteria	Firmicutes	Clostridia	Clostridiales	Ruminococcaceae	Oscillibacter	NA
ASV568	0.054532471	0.059692501	Bacteria	Firmicutes	Clostridia	Clostridiales	NA	NA	NA
ASV379	0.055955472	0.061054862	Bacteria	Firmicutes	Clostridia	Clostridiales	Lachnospiraceae	NA	NA
ASV47	0.058091276	0.063229571	Bacteria	Firmicutes	Clostridia	Clostridiales	Ruminococcaceae	NA	NA
ASV96	0.064879021	0.070444623	Bacteria	Firmicutes	Clostridia	Clostridiales	NA	NA	NA
ASV2507	0.065388918	0.070824671	Bacteria	Firmicutes	Clostridia	Clostridiales	NA	NA	NA
ASV1715	0.075888147	0.081996217	Bacteria	Firmicutes	Erysipelotrichia	Erysipelotrichales	Erysipelotrichaceae	NA	NA
ASV2088	0.079209799	0.085376985	Bacteria	Firmicutes	Clostridia	Clostridiales	Ruminococcaceae	Hydrogenoanaerobacterium	NA
ASV532	0.080523074	0.086581848	Bacteria	Firmicutes	Clostridia	Clostridiales	Ruminococcaceae	Ethanoligenens	NA
ASV552	0.084369908	0.090498472	Bacteria	Firmicutes	Clostridia	Clostridiales	Ruminococcaceae	Sporobacter	NA
ASV800	0.085859046	0.091873327	Bacteria	Firmicutes	Clostridia	Clostridiales	NA	NA	NA

Abbreviations: ASV, amplicon sequence variant; BH-FDR, Benjamini-Hochberg false discovery rate correction.

Notes: ASVs shown where the BH-FDR p-value < 0.1. P-values obtained from a likelihood ratio test of nested models fit via DESeq2 version 1.24.0.
